# Integrating Mendelian randomization, machine learning and retrospective clinical data: an exploratory analysis of the cross-disease association between CHB and PD, with a focus on eosinophil alterations

**DOI:** 10.3389/fneur.2026.1819000

**Published:** 2026-07-09

**Authors:** Yao Ge, Hongbin Cai, Yike Li, YaTing Li, HuiFang Liu, Guilin Zeng, Kui Yang, Yang Luo

**Affiliations:** 1 The First School of Clinical Medicine, Lanzhou University, Lanzhou, China; 2 Department of Neurology, The Second Hospital of Lanzhou University, Lanzhou, Gansu, China; 3 Department of Neurology, The First Hospital of Lanzhou University, Lanzhou, Gansu, China; 4 Key Laboratory of Biotherapy and Regenerative Medicine, The First Hospital of Lanzhou University, Lanzhou, Gansu, China

**Keywords:** CHB (chronic hepatitis B), eosinophil, machine learning, Parkinson’s disease, phenylalanine metabolism

## Abstract

**Background:**

Epidemiological studies on the association between chronic hepatitis B (CHB) and Parkinson’s disease (PD) have yielded inconsistent findings, with causality obscured by confounding and limited mechanistic evidence.

**Methods:**

The study employed an integrated approach encompassing two-sample Mendelian randomization(MR), multi-omics analysis, machine learning-driven gene screening, immune infiltration profiling, and multicenter retrospective clinical validation, with retrospective clinical validation conducted in two independent cohorts.

**Results:**

MR provided evidence suggesting a genetically predicted inverse association between susceptibility to CHB and the risk of PD (OR = 0.82–0.94, *p* < 0.05). Through the integration of machine learning and multi-omics data, *RTN3* and *MAP4K3* were recognized as priority cross-disease genes linking these two conditions. Phenylalanine metabolism emerged as an amino acid pathway showing consistent dysregulated patterns between the two diseases, with peripheral phenylalanine levels exhibiting a divergent trend: elevated in CHB while relatively lower in PD. Immune infiltration analysis and clinical hematological data suggested that eosinophil levels tended to decline in CHB but rise in PD, and such divergent expression patterns may be linked to the observed inverse correlation between CHB and PD susceptibility. (OR = 8.99, *p* < 0.001).

**Conclusion:**

Genetic susceptibility to chronic hepatitis B is inversely associated with Parkinson’s disease risk. The shared pathophysiological landscape potentially involves *MAP4K3, RTN3*, phenylalanine metabolism, and eosinophil. These factors represent candidate therapeutic targets and peripheral biomarkers for PD risk reduction.

## 1 Introduction

Parkinson’s disease (PD) is the second most prevalent neurodegenerative disease worldwide (1). Epidemiological projections indicate that by 2050, the global number of individuals living with PD will surge to 25.2 million, representing a 112% increase from 2021 (2). The core pathological hallmark is the progressive degeneration and loss of dopaminergic neurons in the substantia nigra pars compacta, accompanied by the abnormal accumulation of Lewy bodies (LBs) and age-related white matter alterations (3, 4). Chronic hepatitis B (CHB), caused by persistent infection with the hepatitis B virus (HBV), affects approximately 254 million people worldwide as of 2022 and remains an unresolved major public health challenge globally (5).

The potential epidemiological link between these two diseases, involving distinct organ systems, has attracted research attention but yielded inconsistent conclusions. Some large-scale observational studies have suggested an increased risk of PD in patients with HBV infection (6, 7), while others have reported a null association (8). However, CHB patients are often comorbid with conditions such as hepatic encephalopathy and liver cirrhosis, which can impair neurological function (9). Nucleoside/nucleotide analogs (NUCs) therapy in CHB patients has been associated with a time-varying reduction in PD risk, highlighting the confounding impact of treatment (8). Similarly, studies on hepatitis C virus (HCV) infection present a complex picture, with some indicating an increased PD risk (10) and others, including those on direct-acting antivirals (DAAs), showing no significant association (11, 12). These discrepancies underscore the limitations of conventional observational studies, which are susceptible to reverse causation, residual confounding (e.g., by comorbidities like hepatic encephalopathy or cirrhosis), and the modulating effects of interventions. Critically, the nature of the association between CHB and PD, as well as the underlying molecular mechanisms governing any cross-organ interaction between the two conditions, remain fundamentally unclear.

Some studies provides a crucial framework for investigating such systemic interactions, positing that the liver can influence central nervous system homeostasis through peripheral mediators including metabolites, inflammatory cytokines, and immune cells (13, 14). Preliminary evidence supports this concept in neuroinflammation; for example, HBV infection has been shown to dysregulate cerebral immune responses and microglial function (15). Studies by Tsai et al. have demonstrated that HBV-induced chronic inflammation can affect cognitive function by regulating CD33-related single nucleotide polymorphisms (SNPs) (16). However, current research investigating the pathways linking CHB to the occurrence and progression of PD remains remarkably scarce.

Therefore, this study systematically integrated transcriptomic data from GEO, metabolomic profiles from MetaboLights, GWAS data from the IEU OpenGWAS platform, and multicenter clinical cohorts. Employing a multidimensional bioinformatics-clinical validation design, the study aimed to explore the potential associations and related molecular signatures between chronic hepatitis B (CHB) and Parkinson’s disease (PD). Specifically, Mendelian randomization (MR) analysis suggested a potential genetic correlation between CHB and a reduced risk of PD. Through weighted gene co-expression network analysis (WGCNA) and machine learning algorithms, *RTN3* and *MAP4K3* were identified as prioritized cross-disease genes across both diseases. Furthermore, metabolomic profiling highlighted phenylalanine metabolism as a differentially regulated pathway. Notably, the study observed a significant reversal in the distribution of eosinophil—a type of immune cell previously unrecognized in PD pathogenesis—between CHB and PD populations, leading to speculate that they may be involved in neuroinflammatory regulation and potentially represent a novel cellular component in liver-brain crosstalk. The overall research workflow is presented in the workflow diagram within the Supplementary materials.

## 2 Materials and methods

### 2.1 Data sources

Gene expression microarray datasets used in this study were obtained from the Gene Expression Omnibus (GEO)^1^, and metabolomic datasets were retrieved from the MetaboLights database^2^. The details of the dataset are shown in the Supplementary material.

### 2.2 Mendelian randomization analysis for causal inference

Genome-wide association study (GWAS) summary statistics for chronic hepatitis B (exposure) and Parkinson’s disease (outcome) were obtained from the IEU OpenGWAS platform^3^. Detailed information regarding the included datasets, including GWAS accession numbers, ancestry backgrounds of the study populations, and sample sizes, is provided in Supplementary Table S1.

Instrumental variables were selected based on the following criteria: Single nucleotide polymorphisms (SNPs) showing significant association with the exposure (*p* < 1 × 10^−5^) were identified; linkage disequilibrium (LD) clumping was performed to remove strongly correlated variants (*r*^2^ < 0.001, with a physical distance threshold of 10,000 kb); and the strength of the instruments was assessed using the F-statistic, retaining only SNPs with an F-statistic ≥10 to mitigate weak instrument bias.

Two-sample Mendelian randomization (MR) analyses were conducted using the TwoSampleMR package in R. The inverse-variance weighting (IVW) method served as the primary approach to estimate the causal effect of chronic hepatitis B on Parkinson’s disease. Secondary analyses, including MR-Egger regression, weighted median, simple mode, and weighted mode methods, were employed to validate the robustness of the causal estimates under different model assumptions. A comprehensive suite of sensitivity analyses was performed to evaluate result reliability. Heterogeneity among instrumental variables was assessed using Cochran’s Q test. Potential horizontal pleiotropy was evaluated via the MR-Egger intercept test. Leave-one-out sensitivity analysis was conducted by iteratively excluding one SNP at a time to assess the influence of individual variants on the overall estimate. Furthermore, the MR-PRESSO (Mendelian Randomization Pleiotropy RESidual Sum and Outlier) method was applied, utilizing a significance threshold determined by 1,000 permutations, to perform global pleiotropy tests, identify and correct for outlier SNPs, and assess result distortion.

### 2.3 Integration of multiple datasets and batch effect correction

To ensure comparability across multiple Parkinson’s disease-related datasets, common genes present in all datasets were first identified for subsequent analysis. Batch information corresponding to each sample was recorded. The ComBat algorithm, based on an empirical Bayes framework, was applied to correct batch effects in the merged expression matrix. This approach eliminates systematic batch differences while preserving genuine biological variation across samples. Principal component analysis (PCA) was performed on both pre- and post-correction expression matrices. And Scatter plots were generated using the top two principal components explaining the highest variance, with samples colored by batch and confidence ellipses overlaid, to visually assess the effectiveness of batch effect removal.

### 2.4 Identification of differentially expressed genes and metabolites

Based on the CHB/PD disease transcriptome dataset from the GEO database, differential expression analysis was performed using the limma package in R. Given that this study utilized peripheral blood gene expression profiles rather than lesion tissues (e.g., liver or brain), where biological differences are typically subtle, a composite filtering strategy was adopted to avoid omitting potentially critical regulatory molecules while maintaining the exploratory power of the study. Specifically, multiple testing bias was corrected using the false discovery rate (FDR) method, applying a relatively lenient exploratory threshold (*adj.p* < 0.15) alongside a fundamental statistical significance requirement (raw.*p* < 0.05). Additionally, a biologically relevant cutoff of |log_2_FC| > 0.263 (corresponding to a ~1.2-fold change) was implemented to identify genes with potential biological effects. For differential metabolites, the threshold is set to *adj.p* < 0.05 after FDR correction (Based on MetaboLights database).

### 2.5 Weighted gene co-expression network analysis (WGCNA)

Weighted gene co-expression network analysis (WGCNA) was performed using the WGCNA, limma, and ggplot2 packages in R, with the random seed set to 1,234 to ensure reproducibility(based on the same dataset as the differential expression analysis in 2.4). Initially, inter-sample normalization of gene expression data was conducted using the normalizeBetweenArraysfunction from the limma package. Subsequently, quality control was executed via the goodSamplesGenesfunction to filter out low-quality samples and genes containing missing values. To eliminate interference from outliers, hierarchical clustering was performed on all samples, and static tree cutting (cut height = 20,000, minimum cluster size = 10) was applied to identify and remove outlier samples, retaining only those within the main cluster for downstream analysis.

For network construction, the pickSoftThresholdfunction was utilized to scan soft threshold powers ranging from 1 to 20, identifying the optimal soft threshold (*β*) that satisfied the scale-free network fit criterion. The rationality of the selected *β* was further validated through visual inspection of scale independence and mean connectivity plots. Based on the optimal *β*, an adjacency matrix was constructed and subsequently transformed into a topological overlap matrix (TOM) and its corresponding dissimilarity matrix. Genes were clustered hierarchically based on TOM dissimilarity, and dynamic tree cutting was employed to identify co-expression modules, with a minimum module size set to 50. Module eigengenes were calculated, and modules with highly similar expression patterns were merged using a merging threshold of 0.25, thereby establishing a stable co-expression module architecture.

Finally, Pearson correlation coefficients between module eigengenes and clinical traits were calculated, and their significance was assessed using Student’s t-tests. The resulting raw.*p* were corrected for multiple testing via the false discovery rate (FDR) method. Correlation results and adj.*p* were visualized as a heatmap to intuitively illustrate the association strength between specific modules and phenotypic traits. The modules with significant *adj.p* were selected as effective gene modules for subsequent analysis.

### 2.6 Functional enrichment analysis

Gene Ontology (GO) enrichment including biological processes (BP), cellular components (CC), and molecular functions (MF)—and KEGG pathway analyses were conducted using the “clusterProfiler” package. Gene set enrichment analysis (GSEA) was performed using curated gene sets from the c2.cp.kegg.v7.3.symbols.gmt collection. Enrichment signals were considered significant at *p* < 0.05 (with *p* < 0.1 regarded as suggestive of enrichment).

### 2.7 Machine learning–based identification of disease-relevant signature genes

Machine learning analyses were performed using the glmnet, randomForest, and related packages in R to identify prioritized cross-disease genes involved in the association between chronic hepatitis B (CHB) and Parkinson’s disease (PD). The analytical pipeline was structured as follows: Initially, a LASSO regression model was constructed using the glmnet package to achieve dimensionality reduction via L1 regularization. Ten-fold cross-validation was employed to select the optimal penalty parameter (lambda), with deviance serving as the model performance metric. Genes with non-zero coefficients at lambda.min were extracted as key features, and LASSO coefficient paths along with cross-validation error plots were generated for evaluation. Subsequently, a random forest model was built using the randomForest package. Model performance was assessed using the out-of-bag (OOB) error, and error rate curves were plotted to confirm stability. Gene importance was quantified based on the MeanDecreaseGini index, and high-importance genes were selected after ranking. A bubble plot was utilized to visualize gene importance. The intersection of genes identified by both LASSO and random forest models was defined as potential core regulatory genes. Following this, the dataset was partitioned into training and testing sets at a 6:4 ratio, and the diagnostic efficacy of these genes was evaluated using receiver operating characteristic (ROC) curve analysis.

### 2.8 Construction of prioritized cross-disease genes co-expression networks and functional enrichment

To elucidate the potential regulatory mechanisms and biological pathways involving the prioritized shared genes in the context of the chronic hepatitis B (CHB) and Parkinson’s disease (PD) association, a gene co-expression network was constructed based on the CHB/PD disease transcriptome dataset from the GEO database. Pearson correlation coefficients were calculated using the corrplot package (version 0.92) in R. A composite thresholding strategy was adopted to balance rigor and exploratory power: a stringent threshold (|*r*| > 0.8 and FDR-adjusted *p* < 0.05) was applied initially to identify high-confidence co-expressed genes; if this yielded an insufficient number of genes to construct a robust network, the threshold was relaxed to |*r*| > 0.5 (while maintaining *adj.p* < 0.05) to capture broader potential regulatory relationships.

### 2.9 Immune infiltration analysis

To conduct a preliminary exploration of differentially distributed immune cell subtypes, immune cell infiltration analysis was performed using R software. The CIBERSORT deconvolution algorithm was applied to estimate the relative abundance of 22 immune cell subtypes, based on the gene expression profiles of peripheral blood mononuclear cell (PBMC) samples from patients with CHB or PD. To ensure data quality, immune cell subtypes with infiltration levels of zero in more than 90% of samples were excluded. Zero values resulting from the deconvolution algorithm were imputed using the minimum 1/2 imputation method. Subsequently, the Wilcoxon rank-sum test was applied to compare immune infiltration levels between groups, and *p*-value was adjusted by the FDR method. Furthermore, Pearson correlation analysis was conducted to explore the associations between core genes and specific immune cell subtypes.

For the correlation analysis between priority cross-disease genes and eosinophil (one of the core findings of this study), a two-part robust modeling strategy was further implemented to validate the authenticity and robustness of the identified association, and to exclude potential false-positive results driven by excessive zero-inflated values(Primarily in the PD cohort). Specifically, logistic regression was first performed across the full sample set, where the binary outcome variable was defined as 0 for samples with undetectable eosinophil infiltration (relative abundance = 0) and 1 for samples with quantifiable eosinophil infiltration (relative abundance > 0), to assess the association between target gene expression and the occurrence probability of eosinophil infiltration. Subsequently, beta regression, the standard statistical method for continuous proportional data within the 0–1 interval, was applied solely to samples with quantifiable eosinophil infiltration to validate the correlation between gene expression and eosinophil infiltration abundance, which can effectively eliminate confounding interference from zero-value observations.

### 2.10 Exploration of immune cell alterations in peripheral blood of patients with cross-disease association between CHB and PD

To Exploration eosinophil’ potential role in cross-disease association between CHB and PD, this study adopted a multicenter retrospective design, collecting clinical data from patients treated at the First and Second Affiliated Hospitals of Lanzhou University between January 2017 and January 2025. This clinical analysis was specifically structured to evaluate the divergent immune signatures between the two diseases by comparing independent cohorts of CHB and PD patients. The baseline data tables of participants from the First and Second Affiliated Hospitals of Lanzhou University are shown in Tables 1, 2.

**Table 1:** Comparison of baseline characteristics among study participants (CHB group, PD group, and control group) in The First Affiliated Hospital of Lanzhou University.

	Variables	All subjects	CHB patients	PD patients	Healthy controls	*p*
*n*		1,072	923	84	65	
Gender discrete, %	Female	401	341	26	34	0.021
	Male	671	582	58	31	
Age		58.20	56.00	75.19	67.31	<0.001
Eosinophil count, × 10^9^/L		0.09	0.08	0.20	0.13	<0.001
Eosinophil ratio, %		1.93	1.79	3.06	2.59	<0.001
Basophil count, × 10^9^/L		0.02	0.02	0.02	0.02	0.022
Basophil ratio, %		0.41	0.42	0.39	0.42	0.803
Monocyte count, × 10^9^/L		0.42	0.42	0.44	0.35	0.64
Monocyte ratio, %		7.36	7.47	6.87	6.56	0.029
Alanine aminotransferase, U/L		77.27	86.18	21.21	21.40	0.008
AST ALT ratio, −		1.43	1.41	1.67	1.32	0.016
Total protein, g/L		64.27	63.73	66.97	68.81	0.004
Albumin, g/L		36.48	35.83	39.74	41.64	<0.001
Globulin, g/L		29.03	29.31	27.22	27.26	0.012
Albumin globulin ratio, −		1.33	1.30	1.49	1.56	<0.001
Total bilirubin, μmol/L		54.62	60.64	16.65	17.08	<0.001
Direct bilirubin, μmol/L		26.66	30.31	3.92	3.29	<0.001
Indirect bilirubin, μmol/L		28.24	30.61	12.54	13.77	<0.001

All continuous variables are presented as mean.

**Table 2:** Comparison of baseline characteristics between study participants (CHB group and PD group) in The Second Affiliated Hospital of Lanzhou University.

	Variables	All subjects	CHB patients	PD patients	*p*
*n*		5,254	4,296	958	
Gender discrete, %	Female	1,880	1,447	433	<0.001
	Male	3,374	2,849	525	
Age		54.01	51.16	66.80	<0.001
Eosinophil count, × 10^9^/L		0.09	0.08	0.12	<0.001
Eosinophil ratio, %		0.02	0.02	0.02	<0.001
Basophil count, × 10^9^/L		0.03	0.03	0.03	0.015
Basophil ratio, %		0.00	0.00	0.00	0.534
Monocyte count, × 10^9^/L		0.45	0.45	0.44	0.677
Monocyte ratio, %		0.08	0.08	0.08	<0.001
Glucose, mmol/L		5.72	5.77	5.51	0.002
Urea, mmol/L		5.72	5.65	6.03	<0.001
Creatinine, μmol/L		67.65	68.13	65.46	0.159
Urea creatinine ratio, −		0.09	0.09	0.10	<0.001
Uric acid, μmol/L		283.54	284.41	279.65	0.164
Potassium, mmol/L		3.79	3.78	3.80	0.429
Sodium, mmol/L		138.12	137.84	139.36	<0.001
Chloride, mmol/L		104.09	103.95	104.68	<0.001
Calcium, mmol/L		2.24	2.24	2.25	0.007
Phosphorus, mmol/L		1.03	1.02	1.07	<0.001
Magnesium, mmol/L		0.85	0.84	0.87	<0.001
Alanine aminotransferase, U/L		77.63	90.16	21.44	<0.001
Aspartate aminotransferase, U/L		78.77	90.91	24.31	<0.001
AST ALT ratio, −		1.51	1.52	1.47	0.23
Gamma glutamyl transferase, U/L		80.52	92.18	28.22	<0.001
Alkaline phosphatase, U/L		120.26	128.38	83.84	<0.001
Total protein, g/L		68.23	68.82	65.63	<0.001
Albumin, g/L		37.98	37.72	39.13	<0.001
Globulin, g/L		30.25	31.09	26.49	<0.001
Albumin globulin ratio, −		1.32	1.28	1.52	<0.001
Total bilirubin, μmol/L		36.32	41.08	14.97	<0.001
Direct bilirubin, μmol/L		15.78	18.74	3.44	<0.001
Indirect bilirubin, μmol/L		18.47	20.19	11.29	<0.001
Total cholesterol, mmol/L		3.54	3.47	3.87	<0.001
Triglycerides, mmol/L		1.23	1.22	1.26	0.085
High density lipoprotein, mmol/L		1.01	0.98	1.13	<0.001
Low density lipoprotein, mmol/L		2.30	2.25	2.50	<0.001
Creatine kinase, U/L		129.42	134.82	105.22	0.211
Lactate dehydrogenase, U/L		231.11	240.03	191.08	<0.001
Alpha hydroxybutyrate dehydrogenase, U/L		154.99	160.55	147.47	<0.001
Amylase, U/L		75.35	76.80	66.35	0.008
Homocysteine, μmol/L		18.40	17.76	19.27	0.012
Cholinesterase, U/L		6.16	5.96	7.07	<0.001
Complement C1q, mg/L		169.13	169.47	168.68	0.658
Iron, μmol/L		16.97	17.53	16.21	0.001

All continuous variables are presented as mean.

Differentiated analytical strategies were applied to the two hospitals’ data. For the First Affiliated Hospital, one-way ANOVA and ROC curves were used to compare eosinophil count and percentage differences across three groups. For the Second Affiliated Hospital, a multidimensional approach was employed: univariate logistic regression first assessed the association between eosinophil counts and risks of PD, CHB; restricted cubic spline (RCS) models with 3 quantile knots examined the non-linear dose–response relationship. Both models were fitted with four progressive covariate-adjusted models (gradually incorporating demographic characteristics, inflammatory markers, liver function, and metabolic indicators) to reduce confounding. And relevant sensitivity analysis was conducted.

Detailed information on the inclusion and exclusion criteria, covariate adjustment specifications and sensitivity analysis is provided in the Supplementary material.

### 2.11 Statistical analysis

Statistical analyses were performed using R software (version 4.2.2) and GraphPad Prism 8.0.2 (GraphPad Software Inc., USA). Comparisons between two groups were conducted using the t-test, whereas comparisons among multiple groups were performed using one-way analysis of variance (ANOVA).

## 3 Results

### 3.1 MR analysis suggests a genetically predicted inverse association between CHB susceptibility and PD risk

**Figure 1:**
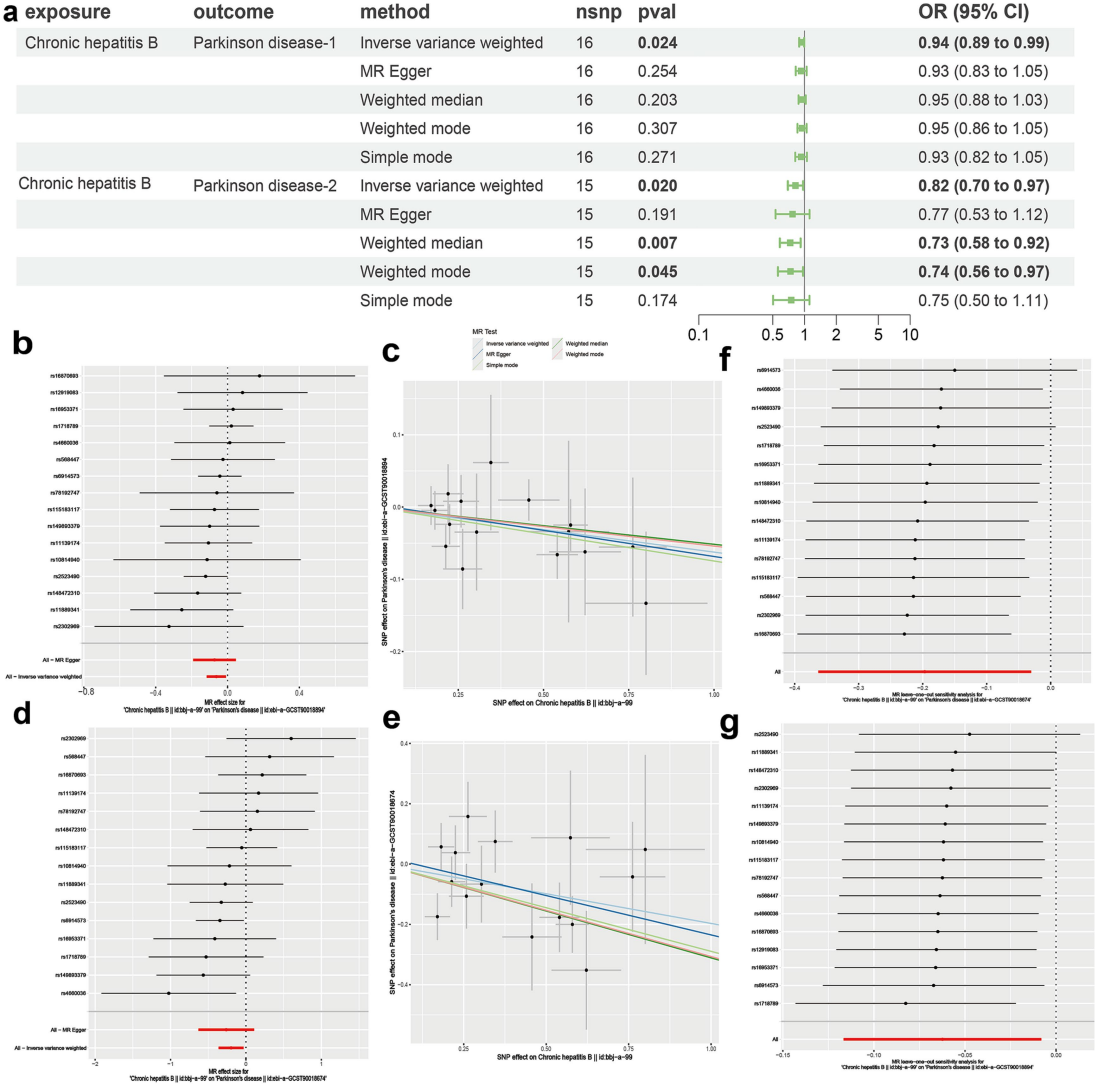
Two-sample Mendelian randomization (MR) analysis of the effect of chronic hepatitis B (CHB) on the risk of Parkinson’s disease (PD). **(a)** Forest plot summarizing the results of two-sample MR analysis with CHB as the exposure and PD as the outcome, based on two independent PD genome-wide association study (GWAS) datasets. The causal effect estimates and statistical significance of the core inverse variance weighted (IVW) method and four supplementary methods are presented. **(b,d)** Forest plots corresponding to the two independent PD GWAS datasets, respectively, showing the individual causal effect estimates and 95% confidence intervals (CIs) of each instrumental variable single nucleotide polymorphism (SNP) on PD risk. **(c,e)** Scatter plots corresponding to the two independent PD GWAS datasets, respectively, visualizing the genetic association between CHB-related genetic instrumental variables and PD outcome. The slope of the fitted line corresponds to the overall causal effect estimate of each MR method. **(f,g)** Forest plots of leave-one-out sensitivity analysis corresponding to the two independent PD GWAS datasets, respectively, showing the overall MR causal effect estimated using the remaining instrumental variables after sequential exclusion of each single SNP. This analysis was performed to verify the stability and robustness of the analysis results.

To elucidate the potential causal association between chronic hepatitis B (CHB) and Parkinson’s disease (PD), two-sample Mendelian randomization (MR) analyses were performed (Figure 1a). Genetically predicted CHB presented a trending association with reduced PD risk (OR = 0.94, 95% CI: 0.89–0.99, *p* = 0.024), and this consistent inverse association was observed in an independent PD dataset (OR = 0.82, 95% CI: 0.70–0.97, *p* = 0.020), which confirmed the cross-cohort robustness of the findings. Complementary MR approaches, including the weighted median method and simple mode method, further verified this trend and strengthened the consistency of the results (Supplementary Tables S2, S3). Forest plots (Figures 1b,d) displayed the effect size of each single nucleotide polymorphism (SNP), while scatter plots (Figures 1c,e) visualized the genetic associations between CHB-related instrumental variables and PD outcomes.

Sensitivity analyses revealed no evidence of horizontal pleiotropy, with non-significant MR-Egger intercepts (PD-GWAS1: 0.004, *p* = 0.85; PD-GWAS2: 0.026, *p* = 0.71), nor significant heterogeneity as determined by Cochran’s *Q* test (PD-GWAS1: 9.16, *p* = 0.86; PD-GWAS2: 15.93, *p* = 0.31) (Supplementary Tables S4, S5). Meanwhile, leave-one-out analysis, in which causal effects were re-estimated after sequential exclusion of each individual SNP, demonstrated no substantial alteration in the direction and significance of the causal effects (Figures 1f,g; PD-GWAS1: *b* = −0.20, *p* = 0.02; PD-GWAS2: *b* = −0.06, *p* = 0.02). The Mendelian Randomization Pleiotropy RESidual Sum and Outlier (MR-PRESSO) test confirmed no significant horizontal pleiotropy (PD-GWAS1: *p* = 0.37; PD-GWAS2: *p* = 0.84), with no outlier SNPs detected, which further supported the reliability of the MR estimates (Supplementary Tables S6, S7). Furthermore, given the potential for population stratification (ancestry heterogeneity) in the datasets used for Mendelian randomization (MR) analysis, additional MR-lap and linkage disequilibrium score regression (LDSC) analyses were performed. The results were consistent with the conclusions presented in this section, and detailed methodologies and complete results are provided in the Supplementary material.

### 3.2 Weighted gene co-expression network analysis (WGCNA) combined with differential analysis identifies key genes for cross-disease association between CHB and PD

Blood serves as the core medium for inter-organ signal transduction between the liver and brain. Transcriptomes of peripheral blood mononuclear cells (PBMCs) from patients with chronic hepatitis B (CHB) and Parkinson’s disease (PD) were analyzed to elucidate the molecular crosstalk between the two conditions. Differential expression analysis was combined with weighted gene co-expression network analysis (WGCNA) for systematic screening of high-dimensional gene expression profiles.

**Figure 2:**
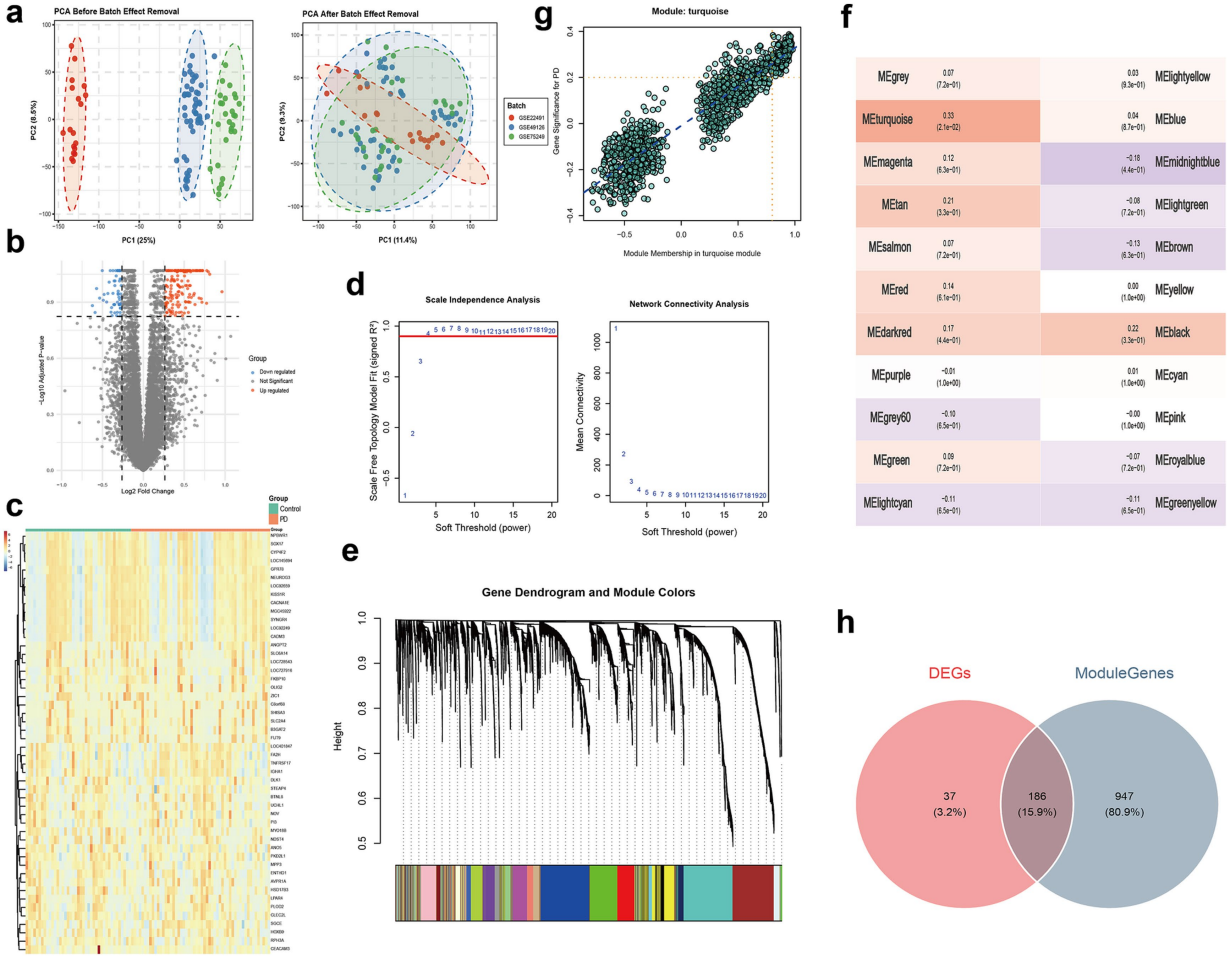
Batch effect correction, differential expression analysis, and weighted gene co-expression network analysis (WGCNA) of PD transcriptomic datasets. **(a)** Principal component analysis (PCA) plots of three integrated PD microarray datasets (GSE22491, GSE49126, GSE75249), showing the sample distribution characteristics before and after batch effect correction. The significantly improved sample clustering after correction indicated favorable homogeneity of the integrated data. **(b)** Volcano plot of differentially expressed genes (DEGs) between the PD group and control group. Upregulated DEGs are marked in red, and downregulated DEGs are marked in blue. Screening thresholds: adjusted *p*-value (*adj.p*) < 0.15, *p* < 0.05, |log₂FC| > 0.263. **(c)** Expression heatmap of the top 50 DEGs. The color depth represents the expression abundance, with red indicating high expression and blue indicating low expression. **(d)** Plot of soft-thresholding power screening in WGCNA, which was used to determine the optimal soft-thresholding power for constructing a gene co-expression network conforming to scale-free topological characteristics. **(e)** Hierarchical clustering tree of genes constructed based on the dissimilarity of the topological overlap matrix (TOM), for the division of distinct co-expressed gene modules. The clustering tree of gene modules is shown at the top, and the corresponding color labels of each module are shown at the bottom. **(f)** Heatmap of the correlation between all gene modules in WGCNA and disease traits. Modules with adj.*p* < 0.05 were defined as disease-significantly associated modules. The color depth represents the strength of correlation, with red indicating stronger positive correlation and blue indicating stronger negative correlation. **(g)** Scatter plot of the correlation between the turquoise module selected for subsequent analysis and disease phenotypes. **(h)** Venn diagram showing the intersection of genes from WGCNA disease-significantly associated modules and DEGs, from which 186 key PD candidate genes were screened.

**Figure 3:**
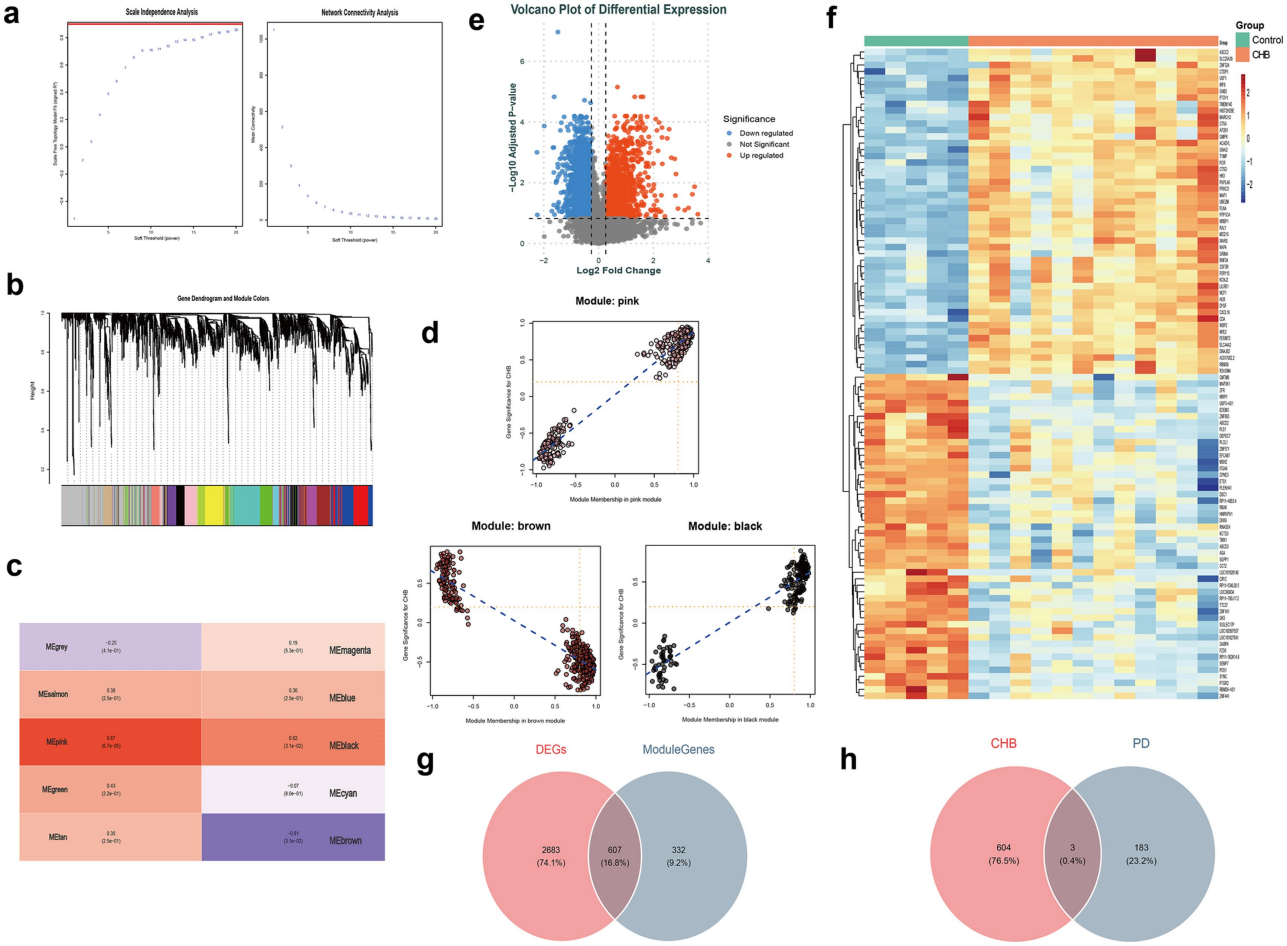
Differential expression analysis, WGCNA, and cross-disease candidate gene screening of CHB transcriptomic datasets. **(a)** Plot of soft-thresholding power analysis in WGCNA, for the construction of a standardized scale-free gene co-expression network. **(b)** Hierarchical clustering tree of genes constructed by WGCNA based on the CHB microarray dataset (GSE58208), showing the division results of gene modules. The clustering tree of gene modules is shown at the top, and the corresponding color labels of each module are shown at the bottom. **(c)** Heatmap of module-trait correlation in WGCNA. Modules with *adj*.*p* < 0.05 were defined as disease-significantly associated modules. The color depth represents the strength of correlation, with red indicating stronger positive correlation and blue indicating stronger negative correlation. **(d)** Scatter plots of the correlation between the pink, brown and black modules selected for subsequent analysis and disease phenotypes. **(e)** Volcano plot of DEGs between the CHB group and control group. Upregulated DEGs are marked in red, and downregulated DEGs are marked in blue. Screening thresholds: *adj.p* < 0.15, *p* < 0.05, |log₂FC| > 0.263. **(f)** Expression heatmap of the top 50 DEGs. The color depth represents the expression abundance, with red indicating high expression and blue indicating low expression. **(g)** Venn diagram showing the intersection of genes from WGCNA disease-significantly associated modules and DEGs, from which 607 key CHB candidate genes were screened. **(h)** Venn diagram of CHB-related candidate genes and PD-related candidate genes, from which 3 shared genes mediating the association between the two diseases were finally screened.

Following batch effect correction, three independent PD transcriptomic datasets were normalized and integrated (Figure 2a). Analysis of the merged cohort identified 224 differentially expressed genes (DEGs), including 165 upregulated genes and 59 downregulated genes. In the CHB dataset, 3,291 DEGs were detected (1,189 upregulated, 2,101 downregulated) (Supplementary Tables S8, S9). Volcano plots highlighted the distribution and statistical significance of upregulated and downregulated DEGs (Figures 2b, 3e), while heatmaps displayed the expression patterns of the top 50 DEGs across samples (Figures 2c, 3f).

To construct scale-free co-expression networks, soft-thresholding powers were selected based on scale independence and mean connectivity: *β* = 4 for the PD dataset and *β* = 7 for the CHB dataset (Figures 2d, 3a). Using these selected thresholds, adjacency matrices were constructed and transformed into topological overlap matrices (TOM), followed by average linkage hierarchical clustering to assign genes with similar expression profiles into distinct modules (Figures 2e, 3b). Ultimately, 22 co-expression modules were identified in the PD dataset, and 10 modules were identified in the CHB dataset. Correlation analyses between co-expression modules and disease phenotypes were subsequently performed (Figures 2f, 3c). Disease-related valid modules for subsequent analyses were screened with the threshold of false discovery rate (FDR)-corrected *p* < 0.05 for module-phenotype correlations. Scatter plots of the correlation between module membership and gene significance of genes within the valid modules are presented in Figures 2g, 3d. For both the PD and CHB datasets, intersection analysis was performed between DEGs identified by differential expression analysis and genes within the valid modules of the corresponding dataset, and disease-specific key gene sets for each of the two conditions were finally obtained (Figures 2h, 3g).

### 3.3 Machine learning algorithms identify *MAP4K3* and *RTN3* as priority cross-disease genes for the cross-disease association between CHB and PD

Intersection analysis of the key gene sets from CHB and PD was performed, and 3 shared candidate genes in peripheral blood for both diseases were prioritized (Figure 3h). To accurately identify priority cross-disease genes that are robustly associated with the phenotypes of both conditions, a dual-algorithm machine learning strategy combining least absolute shrinkage and selection operator (LASSO) regression and random forest (RF) was adopted for screening. LASSO regression achieves feature dimensionality reduction based on L1 regularization, which can eliminate redundant genes without independent disease predictive value and control collinearity among candidate genes. As an ensemble learning algorithm, RF can capture the nonlinear association between genes and disease phenotypes and quantify the contribution of each gene to disease classification. Complementary verification of the two algorithms was performed to reduce the screening bias of a single algorithm.

**Figure 4:**
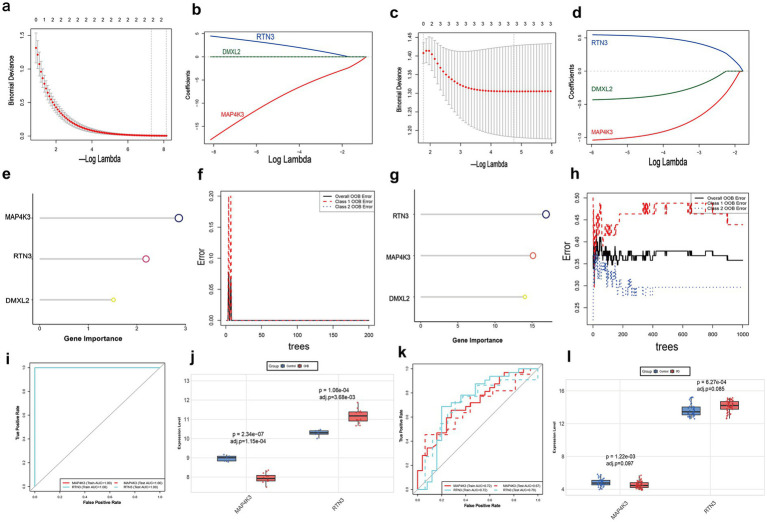
Screening of priority cross-disease core genes linking chronic hepatitis B (CHB) and Parkinson’s disease (PD) based on machine learning algorithms. **(a,b)** Least absolute shrinkage and selection operator (LASSO) regression analysis of key genes in CHB. **(a)** Selection plot of the model tuning parameter *λ*, with the optimal parameter set at *λ* = 0.0002; **(b)** Distribution curve of gene coefficients, in which *DMXL2* was excluded as its coefficient was shrunk to 0. **(c,d)** LASSO regression analysis of key genes in PD. **(c)** Selection plot of the model tuning parameter *λ*, with the optimal parameter set at λ = 0.008; **(d)** Distribution curve of gene coefficients. **(e,f)** Random forest (RF) analysis of key genes in CHB. **(e)** Ranking plot of gene feature importance; **(f)** Change curve of model out-of-bag (OOB) error. **(g,h)** Random forest (RF) analysis of key genes in PD. **(g)** Ranking plot of gene feature importance; **(h)** Change curve of model out-of-bag (OOB) error. By intersecting the results of LASSO regression and random forest analysis, *MAP4K3* and *RTN3* were finally screened as the priority cross-disease core genes mediating the association between CHB and PD. **(i,j)** Box plots of the expression of *MAP4K3* and *RTN3* in the CHB cohort (differential analysis was performed based on the limma package) **(i)**, and receiver operating characteristic (ROC) curves **(j)**. **(k,l)** Box plots of the expression of *MAP4K3* and *RTN3* in the PD cohort (differential analysis was performed based on the limma package) **(k)**, and receiver operating characteristic (ROC) curves **(l)**.

Optimal penalty parameters were obtained by LASSO regression screening (PD cohort: *λ* = 0.008; CHB cohort: λ = 0.0002, Figures 4a,c). All 3 candidate genes in the PD dataset retained non-zero coefficients (Figure 4d), while *DMXL2* in the CHB dataset was eliminated as its coefficient was compressed to 0 (Figure 4b). In addition, RF models (constructed based on all candidate genes) provided supplementary evidence (Figures 4f,h) that *MAP4K3* and *RTN3* had more prominent feature importance in both cohorts compared with *DMXL2*, the redundant gene eliminated by LASSO regression (Figures 4e,g).

Then, the datasets were divided into training sets and test sets at a ratio of 6:4 for further receiver operating characteristic (ROC) curve analysis(Figures 4i,k). The results showed that the area under the curve (AUC) values of *MAP4K3* and *RTN3* were all greater than 0.67 in both the training and test sets, which further verified the potential of *MAP4K3* and *RTN3* with favorable diagnostic efficacy in CHB and PD cohorts identified in the exploratory analysis. In summary, *MAP4K3* and *RTN3* can be prioritized as cross-disease genes associated with the pathogenesis of the CHB-PD association, providing candidate targets and a research basis for further in-depth exploration of the molecular regulatory links between the two conditions.

### 3.4 Co-expression network of priority cross-disease genes and GSEA: revealing the regulatory directions of immune cells and amino acid metabolism

To elucidate the potential mechanistic roles of the priority cross-disease genes identified by LASSO regression and RF in the cross-disease association between CHB and PD, a method combining co-expression network construction via correlation analysis and single-gene Gene Set Enrichment Analysis (GSEA) was used to explore the regulatory functions of *MAP4K3* and *RTN3*. Co-expression networks were constructed from CHB and PD transcriptomic data using Pearson correlation coefficients (PD: |*r*| > 0.5; CHB: |*r*| > 0.8, adjusted *p* < 0.05) (Supplementary Tables S10, S11). Intersection analysis identified 2 genes (*RNF24* and *AHR*) in the *MAP4K3* network, with no significant Gene Ontology (GO) or Kyoto Encyclopedia of Genes and Genomes (KEGG) enrichment. In contrast, 8 genes (*ANP32E*, *CMIP*, *LILRB1*, *NEDD1*, *PTCD2*, *RWDD3*, *SERINC5*, and *ZNF121*) were identified in the *RTN3* network, which were enriched in GO and KEGG pathways (Supplementary Figures S2a,b). GO enrichment highlighted the serine metabolic pathway, while KEGG enrichment pointed to B cell signal transduction. Notably, GSEA of *MAP4K3* also enriched B cell signaling (Supplementary Figure S2d), suggesting a key pathological role of B cells. GSEA revealed trends in amino acid metabolism: GSEA of *RTN3* in PD showed upregulation of glycine, serine and threonine metabolism (Supplementary Figure S2c); GSEA of *MAP4K3* showed near-significant enrichment of phenylalanine metabolism in both PD (normalized enrichment score [NES] = −1.40, *p* = 0.049) and CHB (NES = 1.34, *p* = 0.09), with opposite enrichment directions (Supplementary Figure S2e,f). Therefore, the preliminary findings of the above exploratory analyses indicated that immune cells (especially B cells) and phenylalanine-centered amino acid metabolism may be the key entry points related to *MAP4K3* and *RTN3* with cross-disease differences between CHB and PD, providing guidance for subsequent studies on metabolite mechanisms and immune microenvironment remodeling.

### 3.5 Metabolomic analysis links priority cross-disease genes to the key effector metabolite phenylalanine with opposite expression patterns in CHB and PD

Based on the enriched amino acid metabolic pathways in the transcriptomic data of CHB and PD (Section 3.4), peripheral blood metabolomic datasets of both diseases were retrieved from the MetaboLights database to characterize the amino acid metabolic profiles.

**Figure 5:**
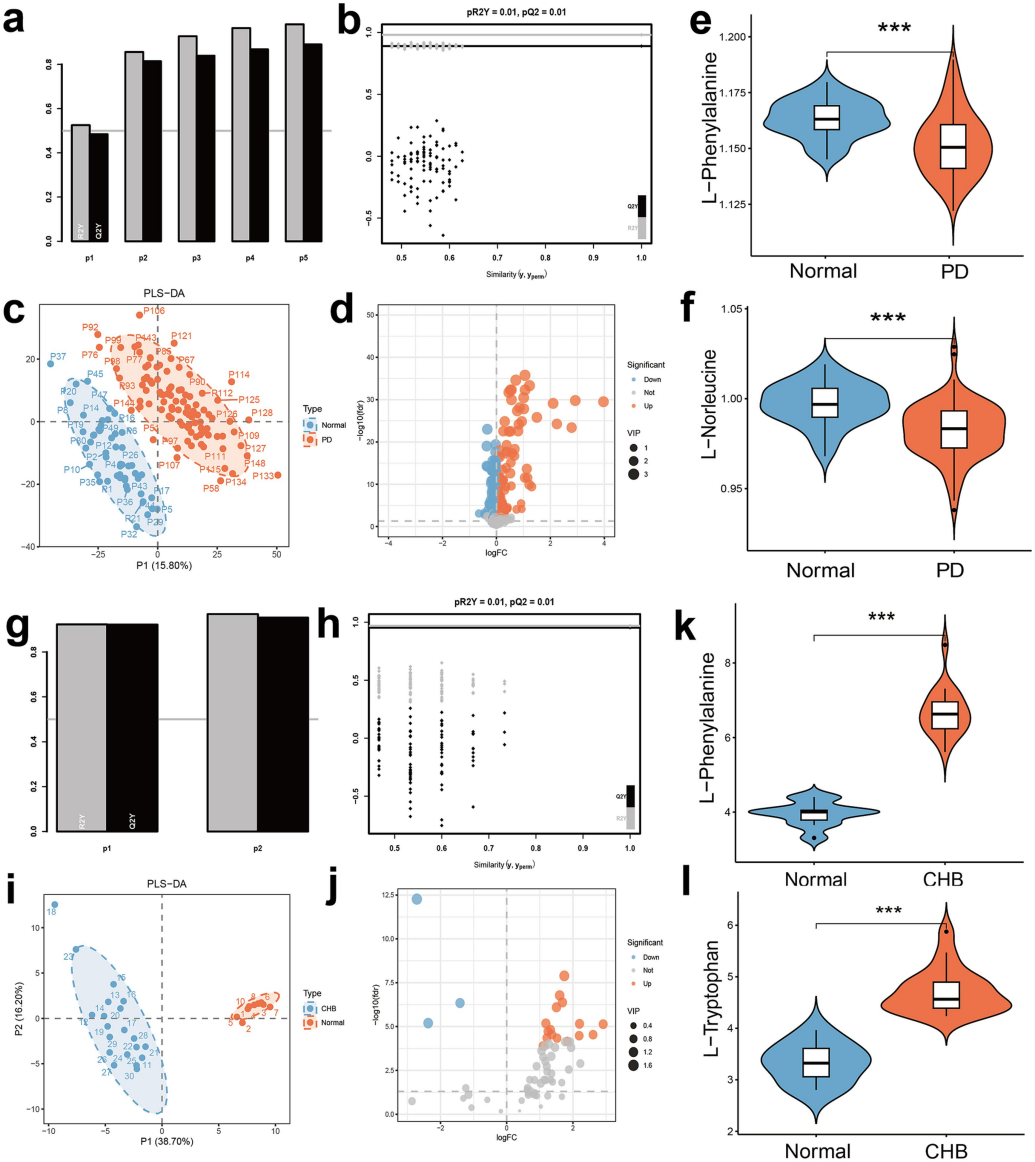
Metabolomic profiling analysis of chronic hepatitis B (CHB) and Parkinson’s disease (PD) (focusing on amino acid metabolic pathways). **(a–c)** Quality control and differential metabolite analysis of the PD metabolomic dataset (MTBLS11094). **(a)** Permutation test distribution plot; **(b)** Metabolite loading plot; **(c)** Partial least squares discriminant analysis (PLS-DA) score plot, showing good separation between the PD group and control group. **(d)** Volcano plot of differential metabolites in the PD group. Upregulated metabolites are marked in red, and downregulated metabolites are marked in blue. Screening threshold: adj.*p* < 0.05. **(e,f)** Box plots of the top 2 differentially expressed amino acids in the PD group. L-phenylalanine **(e)**: log₂FC = −0.02, *adj*.*p* < 0.001, variable importance in projection (VIP) = 1.6; L-norleucine **(f)**: log₂FC = −0.03, *adj.p* < 0.001, VIP = 1.83. **(g–i)** Quality control and differential metabolite analysis of the CHB metabolomic dataset (MTBLS11406). **(g)** Permutation test distribution plot; **(h)** Metabolite loading plot; **(i)** PLS-DA score plot, showing clear clustering and distinction between the CHB group and control group. **(j)** Volcano plot of differential metabolites in the CHB group. Upregulated metabolites are marked in red, and downregulated metabolites are marked in blue. Screening threshold: *adj.p* < 0.05. **(k,l)** Box plots of the top 2 differentially expressed amino acids in the CHB group. L-phenylalanine **(k)**: log₂FC = 2.91, *adj.p* < 0.001, VIP = 1.10; L-tryptophan **(l)**: log₂FC = 1.50, *adj.p* < 0.001, VIP = 1.22. Note: VIP, variable importance in projection. Significance labels: *p < 0.05; **p < 0.01; ***p < 0.001. The differential analyses in Figure **(e,f,k,l)** were all performed using the limma R package.

Partial least squares discriminant analysis (PLS-DA) was performed to verify data reliability. The Q^2^Y of both models was ≥ 0.5, and the permutation test showed *p* < 0.05, confirming robust group separation (Figures 5a,b,g,h). Screening of differential metabolites (*adj.p* < 0.05) identified 1,337 metabolites in PD (415 upregulated, 922 downregulated; Figure 5d) and 73 metabolites in CHB (55 upregulated, 18 downregulated; Figure 5j). Extraction of amino acid metabolites revealed specific alterations in tryptophan and phenylalanine in CHB, while PD showed broader amino acid dysregulation (Supplementary Table S12).

Focusing on metabolites in the pathways enriched by priority cross-disease genes, serine was downregulated only in PD. L-phenylalanine showed bidirectional changes (PD: log_2_FC = −0.02, adjusted *p* < 0.001, VIP = 1.6; CHB: log_2_FC = 2.91, adjusted *p* < 0.001, VIP = 1.10), which was consistent with the GSEA trends (Figures 5e,k). The above results were consistent with the GSEA findings, suggesting that L-phenylalanine is a related metabolite with cross-disease differences between CHB and PD. Its disease-specific expression patterns in CHB and PD provide metabolomic evidence at the liver-brain axis metabolic level for the genetically predicted inverse association between CHB and PD.

### 3.6 Immune infiltration analysis via CIBERSORT reveals disease-specific inverse correlations of eosinophil with priority cross-disease genes and opposite infiltration levels in CHB and PD

**Figure 6:**
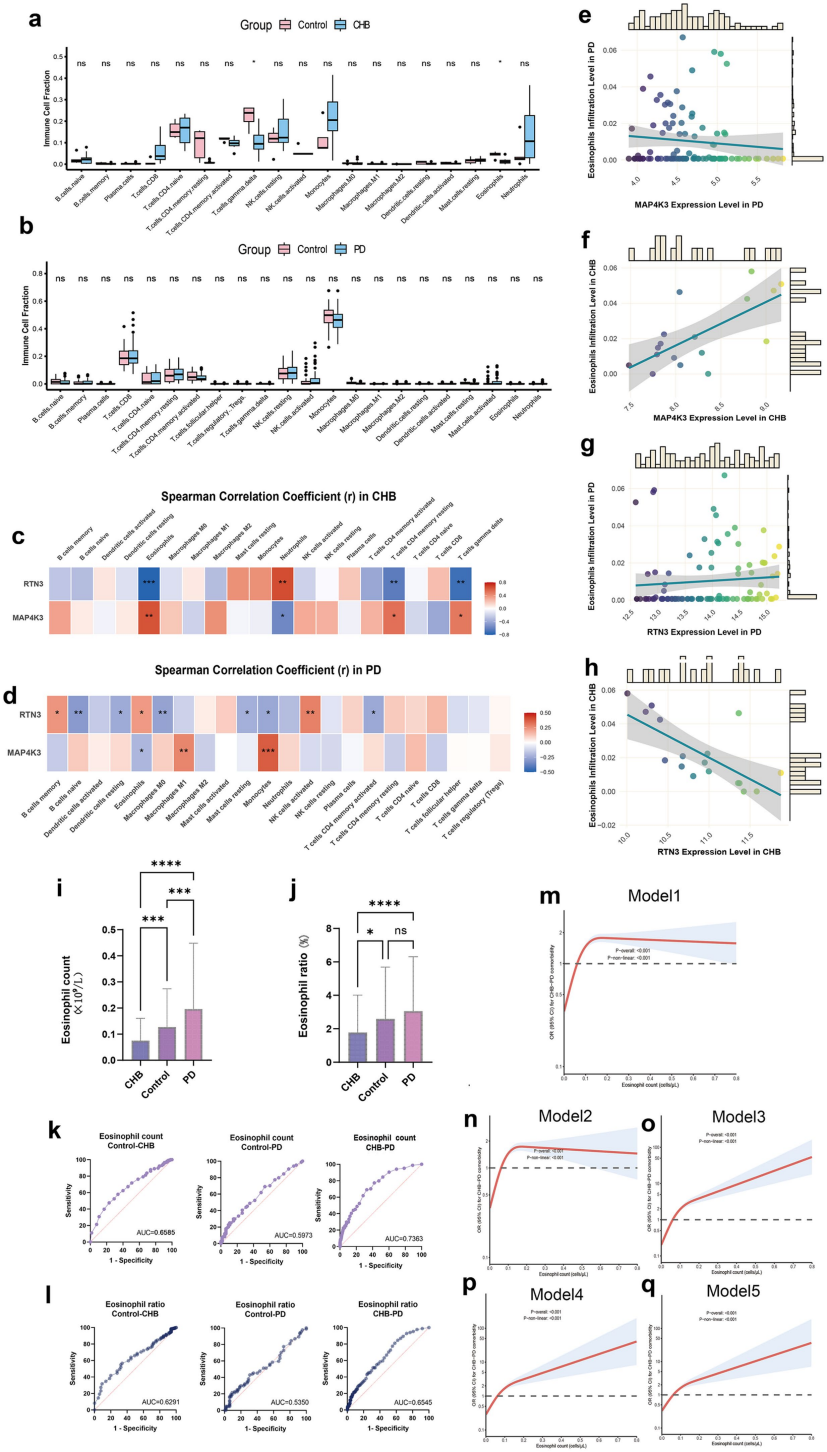
Immune infiltration analysis, correlation between core genes and eosinophil, and clinical validation of eosinophil in CHB-PD association. **(a,b)** Stacked plots of the relative proportion of 22 immune cell subtypes in the CHB cohort **(a)**, PD cohort **(b)** and their corresponding control groups, estimated based on the CIBERSORT deconvolution algorithm. **(c,d)** Heatmaps of the correlation between priority cross-disease core genes (*MAP4K3*, *RTN3*) and immune cell subtypes in the PD cohort **(c)** and CHB cohort **(d)**. The color depth represents the strength of correlation, with blue indicating negative correlation and red indicating positive correlation. **(e,f)** Scatter plots of the correlation between *MAP4K3* expression and the relative proportion of eosinophil in the CHB cohort **(e)**: *r* = 0.632, *p* = 0.0007; and the PD cohort **(f)**: *r* = −0.209, *p* = 0.041. **(g,h)** Scatter plots of the correlation between *RTN3* expression and the relative proportion of eosinophil in the CHB cohort **(g)**: *r* = −0.775, *p* = 0.0004; and the PD cohort **(h)**: *r* = 0.230, *p* = 0.024. **(i)** Results of one-way analysis of variance (ANOVA) for the absolute count of eosinophil in the CHB group, healthy control group and PD group, showing a bidirectional reverse difference among groups (*F* = 49.68, *p* < 0.0001). **(j)** Results of one-way ANOVA for the percentage of eosinophil in the above three groups, with the change trend consistent with that of the absolute count (*F* = 13.52, *p* < 0.0001). **(k,l)** ROC curves of the absolute count **(k)** and percentage **(l)** of eosinophil for intergroup differentiation. **(m–q)** Restricted cubic spline (RCS) curves of the nonlinear dose–response relationship between the absolute count of eosinophil and the risk of CHB-PD association, including 5 stepwise covariate-adjusted models: **(m)** Model 1 (unadjusted); **(n–q)** Model 2 (adjusted for age and gender), Model 3 (adjusted for inflammatory cell-related indicators), Model 4 (adjusted for liver function-related indicators), and Model 5 (adjusted for metabolism-related indicators), respectively. All models showed a significant overall association and nonlinear characteristics between the two variables (all *p* < 0.001). Note: ns, no statistical significance. Significance labels: *p < 0.05; **p < 0.01; ***p < 0.001. The comparisons in Figure **(a,b)** were performed using the Wilcoxon rank-sum test; the intergroup comparisons in Figure **(i,j)** were performed using one-way ANOVA.

To elucidate the interaction between the priority cross-disease genes *MAP4K3* and *RTN3* and immune microenvironment perturbations in the cross-disease association between CHB and PD, 22 immune cell subtypes in PBMC transcriptomes were quantified via CIBERSORT (Figures 6a,b) (Supplementary Table S13). Pearson correlation analysis was performed to explore their regulatory links with specific immune cell subtypes (Figures 6c,d) (Supplementary Table S14). No immune cell subtypes with significant differences were identified in PD after FDR correction of *p* values (Figure 6b), while CHB showed increased CD8^+^T cells and decreased eosinophil levels (Figure 6a) (Supplementary Table S13). There were no significant differences in naive B cells and memory B cells in either disease, which failed to support the findings in Section 3.4.

However, a particularly notable finding was the reversal of the correlation directions between *MAP4K3*/*RTN3* and eosinophil. In CHB, MAP*4K3* was positively correlated (*r* = 0.63, *p* < 0.001) and *RTN3* was negatively correlated (*r* = −0.77, *p* < 0.001) with eosinophil; whereas in PD, the correlation directions were completely reversed (*MAP4K3*: *r* = −0.21, *p* = 0.041; *RTN3*: *r* = 0.23, *p* = 0.024) (Figures 6e–h). Considering the high zero-inflated proportion of eosinophil counts in the PD dataset, a two-part robust regression strategy was further conducted to verify the reliability of these correlations and eliminate zero-value-driven false positives (Supplementary Table S15). The supplementary regression results demonstrated that the priority cross-disease genes exhibited significant or marginally significant associations with eosinophil infiltration status in the full-sample logistic regression (*RTN3*: *p* = 0.015; *MAP4K3*: *p* = 0.062, marginally significant), while neither gene reached a statistically significant correlation in the beta regression restricted to non-zero eosinophil samples (*RTN3*: *p* = 0.056, marginally significant; *MAP4K3*: *p* = 0.149). These results indicated that the expression of our priority cross-disease genes was only associated with the presence or absence of eosinophil infiltration, but had no stable correlation with the abundance of infiltration after eosinophil were detected. Therefore, the Pearson correlation coefficients identified in the primary analysis were not statistically robust, and the relevant correlative results should be interpreted with caution. Nevertheless, combined with the adverse genetic correlation between CHB and PD identified in the previous Mendelian randomization analysis, it is reasonably hypothesized that eosinophil may exhibit inconsistent expression and regulatory patterns during the pathological progression of the two diseases.

### 3.7 Eosinophil count as an independent factor distinguishing CHB and PD phenotypes with group-specific distribution

To verify the findings in Section 3.6, a multicenter retrospective study was conducted based on independent clinical cohorts from the First and Second Affiliated Hospitals of Lanzhou University to explore the potential association between eosinophil levels and distinct phenotypic signatures of CHB and PD. Intergroup comparisons revealed a bidirectional inverse distribution pattern of eosinophil counts and percentages among the three groups. Specifically, eosinophil counts (*p* = 0.001) and percentages (*p* = 0.0242) in patients with CHB were significantly lower than those in healthy controls, and both indicators were also markedly lower in CHB patients than in PD patients (all *p* < 0.0001). In contrast, eosinophil counts in PD patients were significantly higher than those in healthy controls (*p* < 0.0001) (Figures 6i,j). ROC curve analysis demonstrated that eosinophil count had favorable discriminatory efficacy for distinguishing CHB and PD populations (AUC = 0.73), which was superior to eosinophil percentage (AUC < 0.7) (Figures 6k,l). Therefore, eosinophil count was selected as the core independent variable in subsequent correlation analyses. This opposite distribution pattern of decreased levels in CHB and elevated levels in PD was highly consistent with the preliminary hypothesis derived from transcriptomic immune infiltration analysis, preliminarily confirming the cross-disease reverse distribution characteristic of eosinophil between the two disorders.

To clarify the independent association between eosinophil count and disease phenotypes and strictly control potential bias, logistic regression models and restricted cubic spline (RCS) models were constructed for systematic analysis based on an independent validation cohort from the Second Affiliated Hospital of Lanzhou University. A full set of methodological quality control was completed prior to analysis. For potential multicollinearity, variance inflation factor (VIF) diagnosis was performed for all covariates included in the fully adjusted model. The results showed that the VIF of all covariates was <4, which excluded the interference of significant multicollinearity on model estimation (Supplementary Table S19).

Univariate logistic regression analysis based on this validation cohort showed that higher eosinophil count was significantly and positively associated with an increased likelihood of PD relative to the CHB cohort (OR = 8.99, 95% CI: 4.92–16.71, *p* < 0.001). The RCS model further confirmed a significant nonlinear threshold effect between the two variables (global *p* < 0.001, nonlinear *p* < 0.001) (Figure 6m). In the 4 covariate-adjusted models with stepwise inclusion of variables (sequentially controlling for demographic characteristics, inflammatory markers, liver function indicators, and metabolism-related indicators), the positive association between eosinophil count and PD risk remained statistically significant (all *p* < 0.001) (Tables 3, 4). The corresponding RCS analyses also verified the global significance and nonlinear characteristics of the association (Figures 6n–q).

**Table 3:** Logistic regression analysis of the association between eosinophil count and the odds of PD versus CHB across multiple adjusted models.

Term	Model	OR	SE	*p*
Eosinophil_countQ1-reference				
Eosinophil_countQ2	M1	2.29	0.12	<0.001
Eosinophil_countQ3	M1	2.77	0.11	<0.001
Eosinophil_countQ4	M1	3.87	0.11	<0.001
Eosinophil_countQ1-reference				
Eosinophil_countQ2	M2	2.62	0.13	<0.001
Eosinophil_countQ3	M2	2.61	0.13	<0.001
Eosinophil_countQ4	M2	3.83	0.13	<0.001
Eosinophil_countQ1-reference				
Eosinophil_countQ2	M3	2.79	0.14	<0.001
Eosinophil_countQ3	M3	3.07	0.14	<0.001
Eosinophil_countQ4	M3	6.00	0.18	<0.001
Eosinophil_countQ1-reference				
Eosinophil_countQ2	M4	2.27	0.15	<0.001
Eosinophil_countQ3	M4	2.35	0.15	<0.001
Eosinophil_countQ4	M4	3.74	0.19	<0.001
Eosinophil_countQ1-reference				
Eosinophil_countQ2	M5	2.08	0.15	<0.001
Eosinophil_countQ3	M5	2.06	0.15	<0.001
Eosinophil_countQ4	M5	3.19	0.19	<0.001

Model 1: unadjusted; Model 2: adjusted for age and gender; Model 3: adjusted for age, gender, and inflammatory cell indicators; Model 4: adjusted for Model 3 covariates plus liver function indicators; Model 5: adjusted for Model 4 covariates plus metabolic indicators.

**Table 4:** Multivariate logistic regression analysis of eosinophil counts and other covariates associated with the odds of PD versus CHB.

Models	OR (95% CI)	SE	*p*
Model 1			
Exposure. Eosinophil count	8.99	0.31	<0.001
Model 2			
Exposure. Eosinophil count	7.87	0.32	<0.001
Gender discrete Male (female as reference)	0.61	0.09	<0.001
Age	1.14	0	<0.001
Model 3			
Exposure. Eosinophil count	67.67	0.88	<0.001
Gender discrete male (female as reference)	0.65	0.09	<0.001
Age	1.14	0	<0.001
Eosinophil ratio	0.00	4.97	<0.001
Basophil count	21074.11	2.78	<0.001
Monocyte count	0.30	0.24	<0.001
Model 4			
Exposure. Eosinophil count	62.75	0.99	<0.001
Gender discrete male (female as reference)	0.81	0.09	0.028
Age	1.14	0	<0.001
Eosinophil ratio	0.00	5.66	<0.01
Basophil count	64.40	3.04	0.17
Monocyte count	0.33	0.27	<0.001
Alanine aminotransferase	1.02	0	<0.001
Aspartate aminotransferase	0.94	0.01	<0.001
Total bilirubin	0.97	0.01	<0.001
Model 5			
Exposure. Eosinophil count	67.43	1.03	<0.001
Gender discrete male (female as reference)	0.94	0.1	0.534
Age	1.14	0	<0.001
Eosinophil ratio	0.00	5.88	<0.01
Basophil count	6.23	3.14	0.56
Monocyte count	0.36	0.28	<0.001
Alanine aminotransferase	1.02	0	<0.001
Aspartate aminotransferase	0.94	0.01	<0.001
Total bilirubin	0.97	0.01	<0.001
Glucose	0.84	0.03	<0.001
Creatinine	1.00	0	0.02
Total cholesterol	1.20	0.05	<0.001

All continuous variables are presented as mean.

To validate the robustness of the core findings, a comprehensive set of sensitivity analyses were performed based on the validation cohort, including different coding transformations of the exposure variable (S1_Exposure_Z-score, S1_Exposure_Log, S1_Exposure_Tertile), restricted analysis after excluding extreme outliers (S2_Restriction), PSM-matched cohort analysis (S3_PSM), and model validation with the minimal covariate adjustment strategy (S4_Covariate_Strategy) (Table 5). All sensitivity analyses were consistent with the main analysis results, and the association between eosinophil count and diseases remained stable and significant throughout, confirming that the core findings were not affected by analysis strategies, outliers, covariate adjustment methods, or sample selection.

**Table 5:** Integrated results of all sensitivity analyses: sensitivity analyses covered multiple methodological validations: exposure variable transformation adopting Z-score standardization (S1_Exposure_Z-score), log-transformation (S1_Exposure_Log) and tertile grouping (S1_Exposure_Tertile); extreme constraint analysis including age restriction within 18–90 years (S2_Restrict_Age_18–90) and 1%–99% quantile winsorization of eosinophil counts (S2_Restrict_Expo_1–99); PSM matched cohort re-analysis (S3_PSM_Matched); and minimal core covariate adjustment strategy validation (S4_Covariate_Strategy).

Term	Model	OR	*p*
Expo_z	S1_Exposure_Z-score	1.56	<0.001
Expo_log	S1_Exposure_Log	2.32	<0.001
Expo_tertileT2	S1_Exposure_Tertile	1.46	0.0015
Expo_tertileT3	S1_Exposure_Tertile	2.14	<0.001
Expo_continuous_backup	S2_Restrict_Age_18–90	1.47	<0.001
Expo_continuous_backup	S2_Restrict_Expo_1–99	1.47	<0.001
Expo_continuous_backup	S3_PSM_Matched	1.30	<0.001
Eosinophil_countQ1-reference			
Eosinophil_countQ2	S4_Minimal_Adjustment	2.12	<0.001
Eosinophil_countQ3	S4_Minimal_Adjustment	2.10	<0.001
Eosinophil_countQ4	S4_Minimal_Adjustment	2.82	<0.001

Subgroup analysis revealed that the positive association between elevated eosinophil count and increased PD risk was generally consistent across subgroups stratified by age, gender, and CHB disease activity (Table 6). Specifically, the risk elevation of PD in the highest quartile of eosinophil count was more pronounced in individuals aged <65 years, males, and patients with high CHB activity. Notably, a distinct threshold effect was observed in the high CHB activity group: only the top two quartiles of eosinophil count were significantly associated with PD risk, with OR values increasing exponentially (Q3: OR = 3.65, Q4: OR = 8.39), while the second quartile did not reach statistical significance (OR = 1.92, 95%CI: 0.74–4.86, *p* = 0.169). This pattern was fully consistent with the nonlinear dose–response relationship identified in the restricted cubic spline analysis, suggesting a concentration-dependent pathogenic role of eosinophil in CHB-associated PD.

**Table 6:** Results of subgroup analyses.

Term	Model	OR	*p*
Eosinophil_countQ1-reference			
Eosinophil_countQ2	Age_ > =65y	2.18	<0.001
Eosinophil_countQ3	Age_ > =65y	2.46	<0.001
Eosinophil_countQ4	Age_ > =65y	3.45	<0.001
Eosinophil_countQ1-reference			
Eosinophil_countQ2	Age_ < 65y	3.01	<0.001
Eosinophil_countQ3	Age_ < 65y	2.77	<0.001
Eosinophil_countQ4	Age_ < 65y	4.19	<0.001
Eosinophil_countQ1-reference			<0.001
Eosinophil_countQ2	Gender_Male	2.88	<0.001
Eosinophil_countQ3	Gender_Male	2.91	<0.001
Eosinophil_countQ4	Gender_Male	4.67	<0.001
Eosinophil_countQ1-reference			
Eosinophil_countQ2	Gender_Female	2.36	<0.001
Eosinophil_countQ3	Gender_Female	2.35	<0.001
Eosinophil_countQ4	Gender_Female	2.88	<0.001
Eosinophil_countQ1-reference			
Eosinophil_countQ2	CHB_Activity_Low_activity	2.41	<0.001
Eosinophil_countQ3	CHB_Activity_Low_activity	2.37	<0.001
Eosinophil_countQ4	CHB_Activity_Low_activity	3.09	<0.001
Eosinophil_countQ1-reference			
Eosinophil_countQ2	CHB_Activity_High_activity	1.92	0.17
Eosinophil_countQ3	CHB_Activity_High_activity	3.65	<0.001
Eosinophil_countQ4	CHB_Activity_High_activity	8.39	<0.001

Stratified by age (<65 years/≥65 years), gender (male/female), and CHB disease activity (ALT cutoff = 40 U/L), with stratified regression results summarized for each predefined subgroup.

In summary, eosinophil is an immune indicator independently associated with the distinct phenotypic characteristics of CHB and PD. Combined with the Mendelian randomization results, the low eosinophil levels observed in patients with chronic HBV infection may be associated with a reduced risk of Parkinson’s disease.

## 4 Discussion

Interest in viral hepatitis-neurodegenerative disease associations has increased recently. Observational studies link CHB to PD risk, but confounding and unclear mechanisms limit interpretation (6). Mendelian randomization, which uses genetic variants as instrumental variables, addresses these limitations by mitigating confounding biases and reverse causation (17). Based on MR analyses, our study suggests a genetic inverse correlation between CHB susceptibility and PD risk. To overcome the limitation of GWAS in explaining complex trait heritability (18), study integrated multi-omics data to explore the cross-disease pathological mechanisms between CHB and PD. Machine learning approaches screened *MAP4K3* and *RTN3* as top candidate cross-disease genes at the transcriptomic level. On the basis of functional enrichment of these candidate genes, we further investigated differences in amino acid metabolism and immune characteristics between the two diseases, with a focus on phenylalanine metabolism and eosinophil-related profiles. The two biological features exhibited marked divergent patterns in CHB and PD respectively, which may provide potential mechanistic clues for the genetic inverse correlation implied by MR analyses.

*RTN3*, a key reticulon (RTN) family member, is an endoplasmic reticulum (ER)-localized integral membrane protein (19–21). It forms ER-phagy receptor complexes (with *TEX264, CCPG1, SEC62*, etc.) to maintain ER homeostasis (22), and assembles the *RTN3–HSPA9–VDAC2* complex for mitochondrial quality control (23). These multifaceted physiological roles provide the structural basis for *RTN3*-mediated intercellular signal regulation. In viral infections, flaviviruses, SV40, and other viruses exploit *RTN3* to remodel membranes, promote ER curvature, and preserve membrane integrity for replication (24, 25). Notably, hepatitis C virus (HCV) upregulates RTN3 expression during infection. Its C-terminal domain scaffolds viral-host interactions to regulate multivesicular body maturation and exosomal viral release. Notably, both *RTN3* isoforms (*RTN3*L&S) are elevated in HCV-infected hepatocytes and peripheral blood (26), consistent with our observations. In the nervous system, *RTN3* supports CNS development, synaptic plasticity, and axonal regeneration and functional recovery post-injury (27, 28). It also regulates neurodegeneration: in Alzheimer’s disease (AD), *RTN3* overexpression reduces BACE1 axonal transport and terminal enrichment, limiting aberrant APP cleavage and A*β* deposition; whereas its deficiency elevates BACE1 levels and exacerbates A*β* accumulation, underscoring neuroprotective potential (29–32). Based on the above studies, we hypothesize that in CHB and PD association, CHB sustains activation of host immune and metabolic pathways to upregulate peripheral *RTN3* expression. Elevated *RTN3* may enhance peripheral immune cell function by maintaining mitochondrial homeostasis and regulating autophagy. It may also cross the blood–brain barrier into the CNS via exosomes, preserving neural ER and mitochondrial integrity and reducing pathological protein aggregation. These mechanisms may collectively attenuate PD-related dopaminergic neurodegeneration, providing a plausible molecular basis for the inverse association with CHB.

Mitogen-activated protein kinases (MAPKs) are a conserved eukaryotic serine/threonine kinase family regulating cell proliferation, differentiation, apoptosis, and immune responses (33). As a key Ste20 subfamily member, *MAP4K3* (germinal center kinase–like kinase) (34) acts as an upstream regulator, phosphorylating downstream kinases (e.g., MAP2Ks) to initiate cascades that activate canonical MAPK pathways (35), playing an essential role in cellular signaling networks. Substantial evidence links MAPK signaling—particularly the p38-MAPK pathway—to the pathogenesis of both CHB and PD. In CHB, viral components including HBx protein and HBe antigen specifically activate p38-MAPK (36, 37). This activation mediates host antiviral immunity by promoting Th1 differentiation and enhancing CTL activity, shaping immune responses and disease progression (37). The pathway is equally pivotal in PD: *α*-SynN103/tauN368 pre-formed fibrils (PFFs) induce aberrant α-synuclein (α-Syn) phosphorylation, leading to excessive p38-MAPK activation and subsequent axonal transport impairment (38). Moreover, persistent p38-MAPK activation can drive sustained stimulation of the downstream NF-κB signaling cascade—a regulatory axis shown to be a key pathogenic driver in chronic CHB infection (39) and in PD (40). Collectively, these findings suggest that the p38-MAPK signaling pathway may represent a potential molecular link between CHB infection and the shared pathological landscape of PD, offering a mechanistic framework for understanding their cross-organ pathological connections.

Pathway analysis of the top candidate cross-disease genes associated with CHB and PD revealed an enrichment trend in the phenylalanine metabolic pathway. From the perspective of differential disease characteristics, we further investigated peripheral metabolomic datasets from patients with CHB and PD. Although this pathway only achieved nominal enrichment significance in the CHB transcriptomic analysis, its consistent bidirectional changes across transcriptomic and metabolomic datasets support it as a promising metabolic mediator in the liver-brain axis. Metabolomic profiling showed phenylalanine upregulated in CHB and downregulated in PD—consistent with clinical evidence from Gao (41) and Hirayama (42), who independently reported elevated serum phenylalanine in CHB patients and reduced levels in PD patients. Mechanistically, chronic immune activation and inflammation-induced oxidative stress impair phenylalanine hydroxylase (PAH), increasing phenylalanine/tyrosine ratios (as seen in HIV-1 infection (43) and HCV patients receiving IFN-α therapy (44)). In PD, phenylalanine and its derivatives (e.g., chromium-D-phenylalanine, 4-trifluoromethyl-(E)-cinnamoyl-L-4-F-phenylalanine) play key neuroregulatory roles: MR analyses link phenylalanine to PD risk genetically, while such derivatives exhibit neuroprotective properties (45–47). Proposed mechanisms include activation of the PI3K/AKT/CREB pathway, which upregulates BDNF and VEGF to enhance cerebral blood flow, neurogenesis, and cognitive function (48). Additionally, elevated phenylalanine also suppresses inflammation by modulating macrophage MAPK signaling or blocking TLR4–NF-κB activation (49), linking phenylalanine metabolism to MAPK signaling. Collectively, these findings imply a potential two-tier mechanistic association. In CHB patients, upregulated MAP4K3, an upstream activator of the MAPK signaling pathway, may drive phenylalanine metabolic reprogramming and further alter circulating phenylalanine levels. The altered circulating phenylalanine profile observed in CHB exhibits a potential inverse correlation with PD susceptibility. This relationship may be partially mediated by plausible biological processes including enhanced neuronal DNA repair and mitigated neuroinflammation, which provides a plausible metabolic perspective to interpret the observed inverse association between CHB and PD.

Immune infiltration analysis combined with clinical dataset validation suggested that eosinophil may serve as a potentially pivotal immune component underlying biological differences between CHB and PD. Beyond their known roles in antiparasitic immunity and allergy, eosinophil act as potent immunomodulators: they trigger NK-cell activation, support B-cell survival/proliferation/antibody secretion (50),and regulate Th1/Th2 polarization (51, 52). Based on these observations, this study proposes that eosinophil could serve as important immune regulatory factors in the peripheral biological interaction network between CHB and PD, and may be correlated with the observed inverse susceptibility pattern across the two diseases. Eosinophil are well-studied in respiratory viral infections but poorly defined in hepatitis and neurodegenerative diseases. The antiviral functions in Eosinophil include viral capture and reduced infectivity (53), assistance in CD8^+^T-cell-mediated anti-influenza responses (54),and IFN-*γ*-enhanced antiviral activity via TLR7 upregulation (55). Hepatitis-related evidence is limited to HCV-associated periportal eosinophil infiltration (correlating with steatosis and fibrosis) (56) and mitigation of hepatic ischemia–reperfusion injury via ST2-dependent IL-13 signaling (57). Eosinophil–neural interactions remain largely unexplored, though they express neuropeptides/receptors, interact with peripheral nerves, and secrete neuroprotective trophic factors (58). While the exact biological interplay connecting eosinophil to CHB and PD remains largely unclarified, we put forward a plausible mechanistic hypothesis. In CHB, eosinophil participate in hepatic antiviral defense, accumulate in liver tissue, and may subsequently undergo functional exhaustion and population decline. Existing studies have demonstrated that eosinophil can regulate systemic inflammation through T-cell interaction or exosome release (57–59). Based on this evidence, we speculate that the altered circulating eosinophil profile in CHB may be linked to perturbations in the liver–brain axis. Such eosinophil changes could potentially correlate with the modulation of neuroinflammatory responses, providing a plausible biological interpretation for the observed inverse correlation between CHB and PD susceptibility.

Despite the multi-omic evidence and clinical validation provided, several limitations in this study warrant consideration. First, the transcriptomic screening threshold adopted in this omics analysis was relatively lenient, with adjusted *p* < 0.15. This loose threshold was intentionally set for exploratory screening to capture potential cross-disease candidate genes and reduce false-negative omission, rather than for rigorous confirmatory validation. Therefore, the omics-derived differential genes should be interpreted cautiously as preliminary exploratory biomarkers. Second, the sample size utilized for machine learning screening was relatively limited. Insufficient sample capacity may weaken the generalization ability and stability of the machine learning algorithm, which could introduce potential selection bias and constrain the reliable extrapolation of identified hub genes. Third, from a methodological perspective, we acknowledge the inherent conceptual and numerical distinctions between transcriptomic-inferred immune cell proportions and clinical absolute counts; however, the cross-platform consistency of biological trends (i.e., divergent eosinophil dynamics between CHB and PD) corroborates the robustness of our core findings. Fourth, regarding clinical validation, while our retrospective analysis provides valuable real-world context for our multi-omics findings, these data serve primarily as supportive observational evidence rather than a definitive causal demonstration. Despite utilizing rigorous propensity score matching (PSM) and multiple sensitivity analyses to mitigate overt baseline imbalances—such as the inherent age discrepancy between CHB and PD cohorts—the potential for residual confounding inherent in retrospective designs warrants consideration. Specifically, three domains of unmeasured covariates regarding systemic eosinophil dynamics may contribute to the observed variance. Age intrinsically influences systemic eosinophil function and basal inflammatory tone, and while statistically adjusted in our models, the complex nuances between physiological immune exhaustion from chronic HBV infection and typical age-related neuro-immune remodeling in PD may not be fully captured by statistical alignment alone. Additionally, detailed histories of nucleos(t)ide analogs (NUCs) were unavailable in our records. Given that NUCs can modulate systemic immune homeostasis and may influence neurodegenerative susceptibility over time, this unmeasured covariate could act as a potential effect modifier in our analysis. And unrecorded environmental factors with immunomodulatory roles, such as smoking, may also exert parallel effects on both PD risk and eosinophil levels. Furthermore, the lack of explicit stratification of CHB progression and PD severity limits our current ability to pinpoint the precise early intervention window where eosinophil dynamics most accurately reflect neuro-immune crosstalk. Fifth, the proposed liver–brain immunoregulatory mechanism remains preliminary. Due to the lack of *in vitro* or *in vivo* functional assays, the causal role of specific candidate genes (*MAP4K3, RTN3*) and eosinophil-mediated interactions stays hypothetical. Finally, the single-region nature of our cohort without ethnic stratification may limit the global generalizability of these results. Nevertheless, the cross-cohort consistency of the eosinophil trends observed in our clinical validation strongly echoes the preliminary hypotheses generated by our omics approach, providing a robust foundation for future prospective investigations and experimental clarifications to substantiate the cross-organ pathways linking CHB and PD.

## 5 Conclusion

By integrating genetic, transcriptomic, and clinical evidence, this study identifies a potential inverse association between chronic hepatitis B and Parkinson’s disease risk. We suggest that this cross-organ relationship is characterized by a shared molecular signature involving *MAP4K3* and *RTN3*, which potentially converges on phenylalanine metabolism and manifests clinically through divergent eosinophil dynamics. Collectively, these findings provide a multi-dimensional framework that nominates candidate pathways for further investigation into PD risk-reduction strategies.

## Data Availability

The original contributions presented in the study are included in the article/Supplementary material, further inquiries can be directed to the corresponding author.

## Footnotes

1
https://www.ncbi.nlm.nih.gov/geo/

2
https://www.ebi.ac.uk/metabolights/

3
https://opengwas.io/

## Supplementary material

SUPPLEMENTARY TABLE S1 Detailed information of included GWAS datasets for Mendelian randomization analysis, including OpenGWAS ID, publication year, population ancestry, disease trait, genome build, total sample size, case number, control number and SNP count.

SUPPLEMENTARY TABLE S2 Instrumental variables screened and adopted for Mendelian randomization analysis between CHB and PD.

SUPPLEMENTARY TABLE S3 Overall results of Mendelian randomization causal analysis between CHB and PD.

SUPPLEMENTARY TABLE S4 Horizontal pleiotropy test results of Mendelian randomization analysis for CHB and PD.

SUPPLEMENTARY TABLE S5 Heterogeneity test results of Mendelian randomization analysis for CHB and PD.

SUPPLEMENTARY TABLE S6 Leave-one-out cross-validation results of Mendelian randomization analysis.

SUPPLEMENTARY TABLE S7 Sensitivity test results of Mendelian randomization based on the MR-PRESSO algorithm.

SUPPLEMENTARY TABLE S8 Detailed results of transcriptomic differential analysis between PD and control groups.

SUPPLEMENTARY TABLE S9 Detailed results of transcriptomic differential analysis between CHB and control groups.

SUPPLEMENTARY TABLE S10 Co-expressed genes screened via correlation analysis with the priority cross-disease gene *MAP4K3*.

SUPPLEMENTARY TABLE S11 Co-expressed genes screened via correlation analysis with the priority cross-disease gene *RTN3*.

SUPPLEMENTARY TABLE S12 Differential amino acid metabolites identified by metabolomic profiling in CHB and PD.

SUPPLEMENTARY TABLE S13 Wilcoxon rank-sum test results of immune cell abundance in CIBERSORT immune infiltration analysis.

SUPPLEMENTARY TABLE S14 Correlation analysis results between priority cross-disease genes and immune cell infiltration levels.

SUPPLEMENTARY TABLE S15 Logistic regression and Beta regression results evaluating the associations of priority cross-disease genes with eosinophil infiltration status and infiltration abundance in the PD cohort.

SUPPLEMENTARY TABLE S16 Complete baseline demographic and clinical characteristics of the enrolled clinical cohort.

SUPPLEMENTARY TABLE S17 Full results of logistic regression exploring the association between eosinophil count and PD/CHB risk across multiple adjusted models.

SUPPLEMENTARY TABLE S18 Full results of multivariate logistic regression of eosinophil counts and other covariates related to PD versus CHB susceptibility.

SUPPLEMENTARY TABLE S19 Variance Inflation Factor (VIF) results for multicollinearity detection of all included covariates.

SUPPLEMENTARY TABLE S20 Detailed results of 1:1 nearest neighbor propensity score matching (PSM) analysis for inter-group baseline balance.

SUPPLEMENTARY TABLE S21 Integrated results of all sensitivity analyses and subgroup analyses on the association between eosinophil count and CHB-PD susceptibility. Sensitivity analyses covered multiple methodological validations: exposure variable transformation adopting Z-score standardization (S1_Exposure_Z-score), log-transformation (S1_Exposure_Log) and tertile grouping (S1_Exposure_Tertile); extreme constraint analysis including age restriction within 18–90 years (S2_Restrict_Age_18–90) and 1%–99% quantile winsorization of eosinophil counts (S2_Restrict_Expo_1–99); PSM matched cohort re-analysis (S3_PSM_Matched); and minimal core covariate adjustment strategy validation (S4_Covariate_Strategy). Subgroup analysis (Subgroup) was stratified by age, gender and CHB disease activity (ALT cutoff = 40 U/L), with stratified regression results summarized for each predefined subgroup.
