# Supplementary material for: Integrating Mendelian randomization, machine learning and retrospective clinical data: an exploratory analysis of the cross-disease association between CHB and PD, with a focus on eosinophil alterations
Source: Front Neurol. 2026 Jul 9;17:1819000. doi: 10.3389/fneur.2026.1819000 (PMC13393456; doi:10.3389/fneur.2026.1819000)
Supplement: Supplementary file 1 [file Supplementary_file_1.docx]

**Supplementary Files**

**Methods and Materials**

**1. Data Sources**

This study was based on the peripheral blood transcriptomic and metabolomic data of PD and CHB. The details of the datasets are shown in Table 1 and 2.

**2. Sample Overlap Bias Assessment and Correction for MR** **Analyses**

To rigorously evaluate sample overlap bias in two-sample Mendelian randomization (MR) analyses, this study combined linkage disequilibrium score regression (LDSC) and MR-Lap (MR with Laplacian regression) for bias detection and correction.

For the exposure (chronic hepatitis B, CHB; bbj-a-99) and outcome (Parkinson's disease, PD; ebi-a-GCST90018674) datasets, LDSC analysis was first performed using the R package TwoSampleMR (v0.5.6). Prior to analysis, quality control was implemented on the genome-wide association study (GWAS) summary statistics. Autosomal biallelic single nucleotide polymorphisms (SNPs) were retained, while variants with a minor allele frequency < 0.01 or an information score (INFO) < 0.9 were excluded. LDSC monitored population stratification by calculating univariate intercepts, with a primary focus on estimating the cross-trait intercept (int_crosstrait). This index quantifies residual correlation that cannot be explained by polygenic signals. A significant deviation from 0 indicates sample-level correlation (e.g., sample overlap or cryptic relatedness), whereas a value close to 0 suggests low overlap risk. Notably, if the heritability of the outcome trait is not statistically significant (h² < 2 standard errors [SE]), the genetic correlation coefficient (rg) may not be computable (reported as NaN); however, this does not affect the validity of the cross-trait intercept for assessing sample overlap risk.

To further validate and correct for potential biases, this study performed Laplacian regression using the R package MRlap (v0.1.2). Compared with conventional methods, MR-Lap can simultaneously correct for both sample overlap bias and weak instrument bias, exhibiting superior statistical power in scenarios with potential sample overlap. The analysis first generated standard results, followed by bias correction using the Laplacian framework to obtain adjusted causal effect sizes and their corresponding standard errors. The impact of sample overlap was quantified by comparing effect estimates before and after correction. MR-Egger intercept analysis was additionally performed to detect horizontal pleiotropy, with the interpretation of Egger intercept results taking into account the potential presence of sample overlap. Ultimately, conclusions were drawn based on the combined evidence from LDSC and MR-Lap. If both methods indicated no significant sample overlap and stable effect estimates, the standard results were presented as the primary findings. If significant overlap was detected or the adjusted effect estimates changed substantially, the MR-Lap corrected results were considered the final conclusion.

**3. Eosinophils in the Correlation between Chronic Hepatitis B and Parkinson's Disease: A Retrospective Study**

**3.1 Participants**

This retrospective study collected cases of chronic hepatitis B (CHB) patients, Parkinson's disease (PD) patients, and control cases (healthy subjects or inpatients undergoing treatment for systemic diseases) admitted to the First and Second Affiliated Hospitals of Lanzhou University from January 2017 to January 2025. The aim was to explore the role of eosinophils in the correlation between CHB and PD. Based on established diagnostic criteria, participants were divided into three groups: PD group, CHB group, and healthy control group.

**3.2 Inclusion and Exclusion Criteria**

The diagnosis of PD was independently confirmed by two neurologists with associate chief physician or higher titles, strictly following the UK Parkinson's Disease Society Brain Bank diagnostic criteria^1^, and secondary Parkinsonism was excluded. The diagnosis of CHB required positive hepatitis B surface antigen (HBsAg) for ≥6 months and alanine aminotransferase (ALT)/aspartate aminotransferase (AST) < 2 times the upper limit of normal. All participants completed a complete blood count (CBC) within 24 hours of admission, and the test time was earlier than adjustments to anti-PD drugs or antiviral therapy. During this period, they had no exposure to drugs that might affect granulocytes, and the test indicators included the absolute count and percentage of eosinophils (EOS), basophils (BASO), and monocytes (MONO). Additional inclusion criteria were: Nutritional Risk Screening 2002 (NRS2002) score < 3 (no nutritional risk); if complicated with diabetes, hypertension, or non-functional lacunar infarction, the condition must be stable (controlled by treatment for ≥3 months with no progression); and complete clinical data such as age, gender, disease duration, and liver and kidney function. Healthy controls required normal liver function, negative HBsAg and hepatitis-related antibodies, no chronic inflammatory diseases, malignant tumors, infections, thyroid or kidney diseases, no history of using drugs affecting granulocytes, and NRS2002 score < 3.

The exclusion criteria for all groups were unified as follows: (1) Granulocyte abnormality-related diseases (eosinophilia, leukemia, acute attack of acute allergic diseases); (2) Hematopoietic system diseases (moderate to severe anemia, hemoglobin < 90g/L; platelets < 100×10⁹/L), estimated glomerular filtration rate < 60mL/min/1.73m², abnormal thyroid function, and NRS2002 score ≥3; (3) Active diseases (acute infection, acute hepatitis, liver cirrhosis, hepatocellular carcinoma, New York Heart Association (NYHA) cardiac function class Ⅲ-Ⅳ); (4) Use of glucocorticoids or immunosuppressants within 3 months, or adjustment of anti-PD drugs in PD patients within 1 month; (5) Other neurodegenerative diseases (Alzheimer's disease, multiple system atrophy, etc.); (6) Incomplete clinical data (missing core CBC indicators or key clinical information).

**3.3 Data Collection and Preliminary Analysis**

Based on the above inclusion and exclusion criteria, 923 CHB patients, 84 PD patients, and 65 control cases were collected from the First Affiliated Hospital of Lanzhou University. One-way analysis of variance (ANOVA) was used to initially explore differences in eosinophil levels among the three groups, and Receiver Operating Characteristic (ROC) curve analysis was further employed to evaluate the discriminatory ability of eosinophils for group classification.

A total of 4296 CHB patients and 958 PD patients were collected from the Second Affiliated Hospital of Lanzhou University. Taking advantage of the large data volume, logistic regression analysis was performed to systematically evaluate the effect of eosinophils in the correlation between the two diseases and the impact of confounding factors.

**3.4 Definition and Processing of Variables, and Inclusion of Confounders**

This study clearly defined the core variables and standardized their processing procedures. Disease status (CHB patients vs. PD patients) was set as the binary outcome variable, and healthy individuals were regarded as the reference group. The absolute eosinophil count was defined as the core exposure variable. In the primary analysis, the exposure variable was stratified into four ordinal categories (Q1–Q4) according to population quartiles, with the lowest quartile Q1 served as the reference. The original continuous form of eosinophil count was retained for subsequent robustness validation. Based on clinical pathological characteristics and previous research evidence, four categories of potential confounding factors were enrolled as covariates, including demographic characteristics, inflammatory cell indicators, liver function and CHB disease activity markers, and metabolic-related indicators, which were used for stepwise covariate adjustment in regression models.

Based on the clinical cohort from the Second Affiliated Hospital of Lanzhou University, five gradually adjusted binary logistic regression models were constructed to systematically explore the association between eosinophil count and the risk of CHB and PD. The independent effect size and stability of the exposure were clarified by controlling confounding bias layer by layer. All models adopted binary disease status as the outcome and absolute eosinophil count as the primary predictor, fitted with a binary logistic link function. The association strength was presented as odds ratio (OR) with 95% confidence interval (95%CI).

The setting of five hierarchical models was as follows: Model 1 (M1_Univariate) was a crude univariate model only incorporating absolute eosinophil count without any covariate adjustment; Model 2 (M2_Adjusted_Gender_Age) further adjusted for demographic covariates (gender and age) on the basis of Model 1; Model 3 (M3_Adjusted_Inflammation) additionally included inflammatory cell indicators, including eosinophil percentage, basophil count and monocyte count; Model 4 (M4_Adjusted_Liver) supplemented core liver function markers, namely alanine aminotransferase (ALT), aspartate aminotransferase (AST) and total bilirubin; Model 5 (M5_Fully_Adjusted) was the fully adjusted model, which further incorporated key metabolic indicators including fasting blood glucose, serum creatinine and total cholesterol (Supplementary Table 17, 18).

After model fitting, variance inflation factor (VIF) was calculated for multicollinearity diagnosis among covariates, and VIF＜5 was taken as the criterion for no significant multicollinearity. The likelihood ratio test was applied to evaluate the goodness-of-fit of each model, ensuring the scientificity and rationality of model construction (Supplementary Table 19).

**3.5 Sensitivity Analysis for Robustness Validation**

To eliminate the interference of analytical strategies, extreme outliers, baseline imbalance and covariate selection on primary conclusions, a multi-dimensional sensitivity analysis was performed to verify result robustness.

Firstly, for the coding mode of exposure variable, Z-score standardization (S1_Exposure_Z-score), log-transformation (S1_Exposure_Log) and tertile grouping (S1_Exposure_Tertile) were separately conducted on absolute eosinophil count, and all transformed variables were re-entered into the fully adjusted model to verify the consistency of association direction and statistical significance.

Secondly, for extreme value restriction and age imbalance, samples with extreme age (＜18 years or ＞90 years) were excluded (S2_Restrict_Age_18-90), and 1%–99% quantile winsorization was performed on eosinophil count (S2_Restrict_Expo_1-99). The fully adjusted model was refitted respectively.

Thirdly, to balance baseline characteristics between groups, 1:1 nearest neighbor propensity score matching (PSM) was performed with gender, age, ALT and AST as matching variables, and the caliper value was set to 0.02 (Supplementary Table 20). Standardized mean difference (SMD＜0.1) was used to evaluate baseline balance after matching, and the regression model adjusted for gender ,age, AST and ALT was refitted in the PSM-matched cohort (S3_PSM_Matched).

In addition, a minimal covariate model only adjusted for three core confounders (gender, age and AST) was constructed to verify whether the conclusion was affected by covariate selection strategies (S4_Covariate_Strategy).

**3.6 Subgroup Analysis**

To explore the population heterogeneity of the association between eosinophil count and CHB-PD risk, and to validate the effect modification of CHB disease activity, preset stratified factors were applied for subgroup analysis. Participants were stratified by age (＜65 years vs. ≥65 years＝ and gender (male vs. female). According to the guidelines for the prevention and treatment of chronic hepatitis B, ALT 40 U/L was set as the cut-off value to divide patients into low CHB activity group (ALT＜40 U/L＝ and high CHB activity group (ALT≥40 U/L), with ALT level regarded as a clinical surrogate indicator of liver inflammatory activity. Regression models adjusted for demographic characteristics were fitted in each subgroup to compare the differences of association effect sizes across stratifications. Detailed results of all sensitivity and subgroup analyses are shown in Supplementary Table 21.

**3.7 Statistical Methods**

All statistical analyses were performed using R software (version 4.2.2). Two-tailed tests were adopted for all hypothesis tests with a significance level of α=0.05. A *P* value less than 0.05 was considered statistically significant to ensure the standardization and reproducibility of the analytical workflow.

**Result**

**1** **Results of Sample Overlap Bias Assessment**

The study performed a systematic bias assessment for the Japanese Biobank-derived datasets (bbj-a-99 and ebi-a-GCST90018674) that may have potential sample overlap using two complementary methods: MR-Lap correction analysis and LDSC cross-trait intercept analysis. The results consistently indicated that sample overlap did not exert a substantial impact on the causal inference of this study(The details are shown in Table3).

LDSC analysis revealed that the SNP heritability of the exposure trait (chronic hepatitis B) was 0.0051 (SE=0.0022), which was significantly greater than zero, indicating a detectable genetic basis. In contrast, the SNP heritability of the outcome trait (Parkinson's disease) was -0.0009 (SE=0.0026). Due to the non-significant heritability of the outcome trait, the genetic correlation coefficient could not be calculated (reported as NaN). This is a well-documented phenomenon of this approach and does not compromise the validity of the cross-trait intercept for detecting sample overlap bias. Critically, the cross-trait intercept was 0.0043 (SE=0.0052, *p*≈0.41), which was extremely close to zero and not statistically significant, clearly demonstrating the absence of statistically meaningful sample overlap bias between the two datasets. The univariate intercepts for the two datasets were 0.9890 and 0.9934, respectively, both very close to 1, indicating no significant population stratification bias and good data quality.

MR-Lap analysis showed that the uncorrected original causal effect was statistically significant (*p*=0.0173). After simultaneous correction for both sample overlap bias and weak instrument bias, the effect size was β=-0.1127 (SE=0.0481, *p*=0.0192), which remained statistically significant. The *p*-value for the difference in effect estimates before and after correction was 0.5043, indicating no statistically significant difference in causal effect estimates between the uncorrected and corrected models. Furthermore, the MR-Egger intercept test yielded a result of -0.0451 (*p*=0.7268), with no significant horizontal pleiotropy detected, further supporting the reliability of the results.

Taken together, even if a very small degree of sample overlap exists between the bbj-a-99 and ebi-a-GCST90018674 datasets, its impact on the causal inference of this study is negligible. Combined with the consistent negative association results obtained from two additional independent Parkinson's disease GWAS datasets (ebi-a-GCST007430 and finn-b-G6_PARKINSON) that have no sample overlap with the chronic hepatitis B dataset, the conclusion of MR is robustness.

**Supplementary Figures**


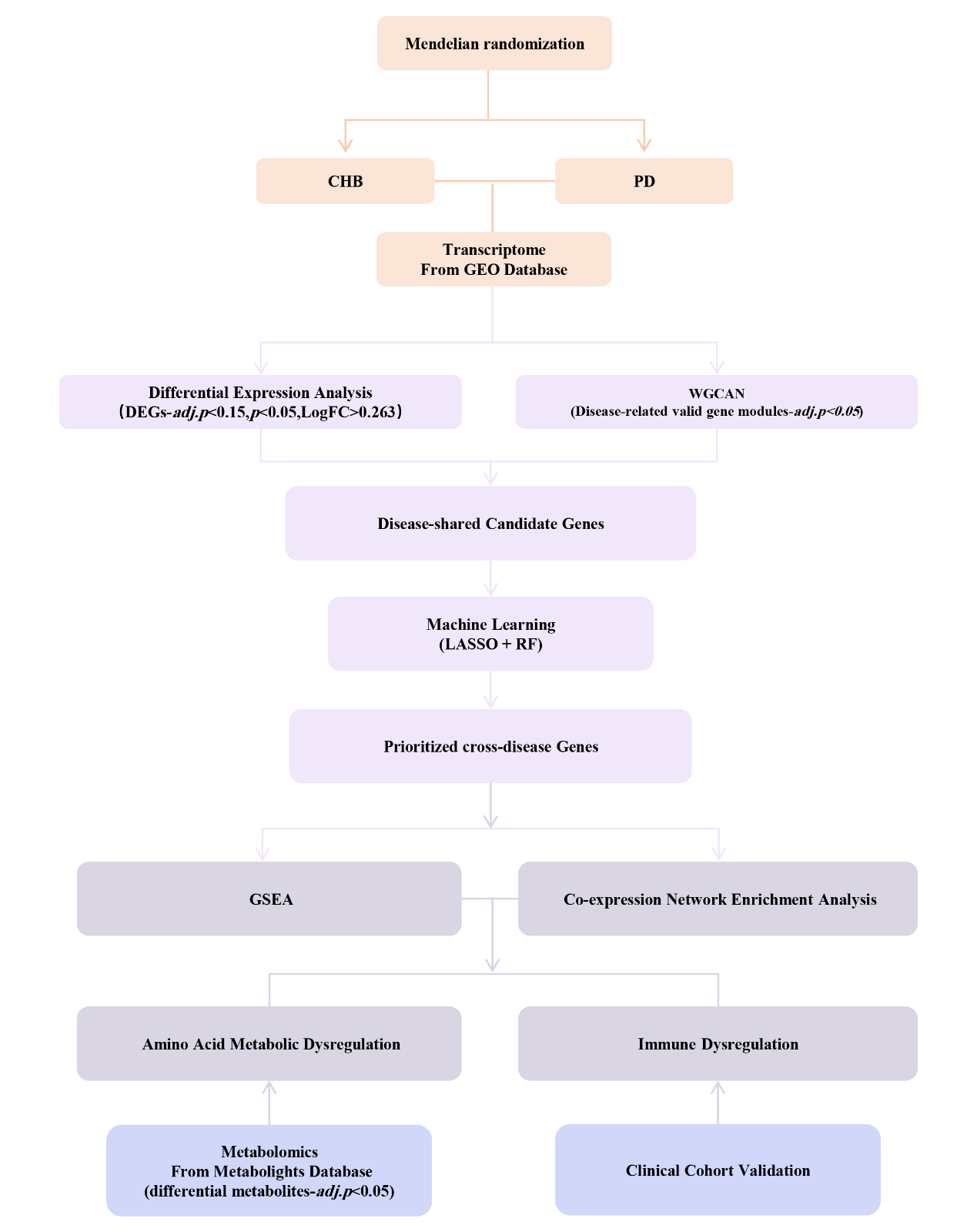


**workflow diagram**

The overall research pipeline starts with two-sample Mendelian randomization (MR) to explore the genetic association between chronic hepatitis B (CHB) and Parkinson’s disease (PD). Transcriptomic datasets from the GEO database are analyzed via differential expression analysis and weighted gene co-expression network analysis (WGCNA) to screen disease-shared candidate genes. Machine learning algorithms are further adopted to identify the prioritized cross-disease genes. Gene set enrichment analysis and co-expression network analysis are then conducted to characterize biological functions related to amino acid metabolic dysregulation and immune dysregulation. Finally, metabolomic profiling and multicenter retrospective clinical cohorts are used to validate the core findings.


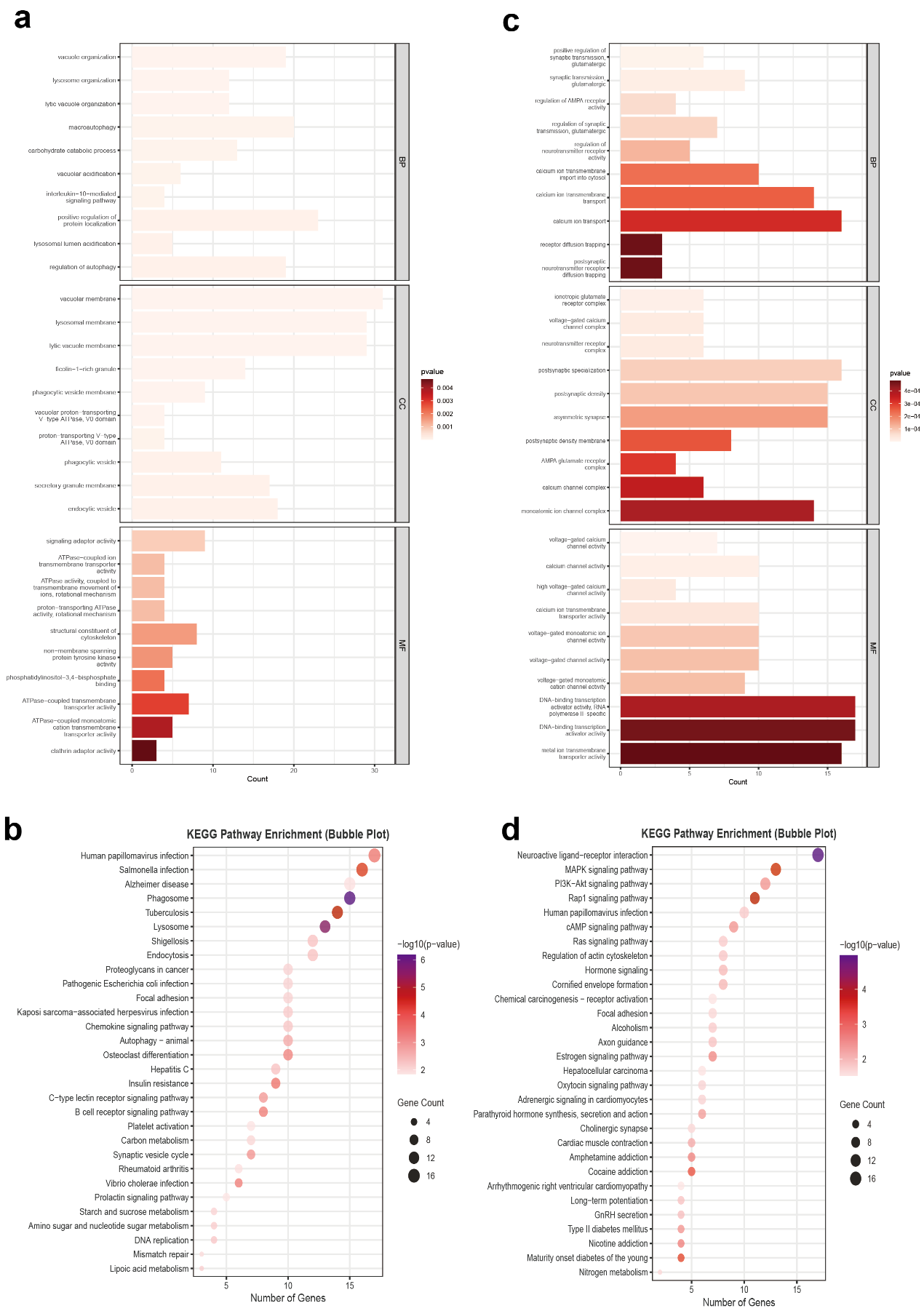


**Supplementary Figure 1Functional enrichment analysis of genes screened from PD and CHB.** (**a**) GO enrichment analysis of genes screened from PD. (**b**) KEGG pathway enrichment analysis of PD-screened genes. (**c**) GO enrichment analysis of genes screened from CHB. (**d**) KEGG pathway enrichment analysis of CHB-screened genes.


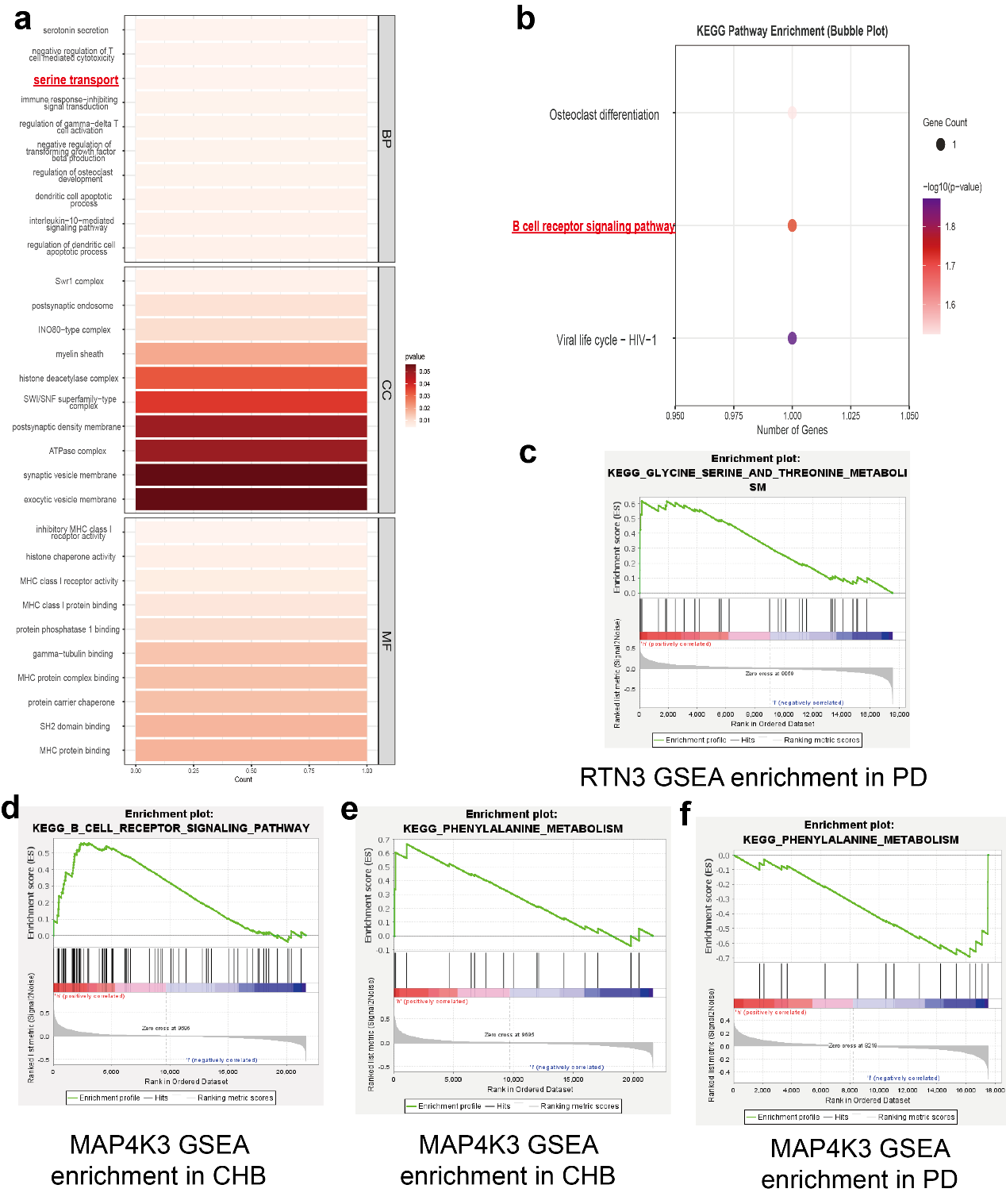


**Supplementary Figure 2 Functional enrichment analysis of core genes and their co-expression networks.** (**a**, **b**) Gene Ontology (GO) (**a**) and Kyoto Encyclopedia of Genes and Genomes (KEGG) (**b**) enrichment analyses of shared genes in the RTN3 co-expression network, highlighting enrichment in serine metabolic pathways and B cell signaling transduction pathways. (**c**) Gene Set Enrichment Analysis (GSEA) of RTN3 in PD, showing significant upregulation of the glycine, serine, and threonine metabolic pathway (NES = 1.42, *p* = 0.022). (**d**) GSEA of MAP4K3 in CHB, indicating enrichment in the B cell signaling transduction pathway (NES = 1.38, *p* = 0.035). (**e**, **f**) GSEA of MAP4K3 showing reciprocal regulation of the phenylalanine metabolic pathway in PD (**e**: NES = -1.40, *p* = 0.049) and CHB (**f**: NES = 1.34, *p* = 0.09).

**References：**

1. Hughes, A. J., Daniel, S. E., Kilford, L. & Lees, A. J. Accuracy of clinical diagnosis of idiopathic Parkinson’s disease: a clinico-pathological study of 100 cases. *J. Neurol. Neurosurg. Psychiatry* **55**, 181–184 (1992).

**Tables**

*Table 1. GEO dataset details*

| Dataset | Platform | Sample Size (Grouping) |
| --- | --- | --- |
| GSE22491 | GPL6480 (Agilent-014850 Whole Human Genome Microarray 4×44K G4112F) | 18 samples: 8 controls, 10 PD |
| GSE49126 | GPL4133 (Agilent-014850 Whole Human Genome Microarray 4×44K G4112F) | 50 samples: 20 controls, 30 PD |
| GSE75249 | GPL4133 (Agilent-014850 Whole Human Genome Microarray 4×44K G4112F) | 20 samples: 13 controls, 7 PD |
| GSE58208 | GPL570 (Affymetrix Human Genome U133 Plus 2.0 Array) | 17 samples: 5 controls, 12 CHB |

*Table 2. MetaboLights dataset details*

| Dataset | Sample Size (Grouping) |
| --- | --- |
| MTBLS11094 | 150 samples: 50 controls, 100 PD |
| MTBLS11406 | 30 samples: 10 controls, 20 CHB |

*Table 3* *Results of Sample Overlap Bias Correction Analysis (bbj-a-99 vs ebi-a-GCST90018674)*

| **Analysis method** | **Parameter** | **Value** |
| --- | --- | --- |
| **MR-Lap Correction Analysis** | Uncorrected effect *p*-value | 0.0173 |
|  | Corrected effect (β) | -0.1127 |
|  | Corrected effect SE | 0.0481 |
|  | Corrected effect *p*-value | 0.0192 |
|  | Test statistic for effect difference | 0.6677 |
|  | *p*-value for effect difference | 0.5043 |
|  | MR-Egger intercept | -0.0451 |
|  | MR-Egger intercept SE | 0.1700 |
|  | MR-Egger intercept *p*-value | 0.7268 |
| **LDSC Analysis** | Exposure heritability (*h*²) | 0.0051 |
|  | Exposure heritability SE | 0.0022 |
|  | Exposure univariate intercept | 0.9890 |
|  | Outcome heritability (*h*²) | -0.0009 |
|  | Outcome heritability SE | 0.0026 |
|  | Outcome univariate intercept | 0.9934 |
|  | Genetic covariance (*g*<sub>cov</sub>) | 0.0013 |
|  | Genetic covariance SE | 0.0016 |
|  | Genetic correlation (*r*<sub>g</sub>) | NaN |
|  | Cross-trait intercept | 0.0043 |
|  | Cross-trait intercept SE | 0.0052 |
| **Genetic Architecture Analysis** | Polygenicity | 6.74×10⁻⁵ |
|  | Per-SNP heritability | 6.62×10⁻⁵ |
